# Superior cellular activities of azido- over amino-functionalized ligands for engineered preQ_1_ riboswitches in *E.coli*

**DOI:** 10.1080/15476286.2018.1534526

**Published:** 2018-10-26

**Authors:** Eva Neuner, Marina Frener, Alexandra Lusser, Ronald Micura

**Affiliations:** a Institute of Organic Chemistry and Center for Molecular Biosciences Innsbruck CMBI, Leopold-Franzens University, Innsbruck, Austria; b Division of Molecular Biology, Biocenter, Medical University of Innsbruck, Innsbruck, Austria

**Keywords:** RNA ligand recognition, modifications, riboswitches, kinetics, thermodynamics, RNA biosensor tools, preQ_1_ derivatives

## Abstract

For this study, we utilized class-I and class-II preQ_1_-sensing riboswitches as model systems to decipher the structure-activity relationship of rationally designed ligand derivatives *in vitro* and *in vivo*. We found that synthetic preQ_1_ ligands with amino-modified side chains that protrude from the ligand-encapsulating binding pocket, and thereby potentially interact with the phosphate backbone in their protonated form, retain or even increase binding affinity for the riboswitches *in vitro*. They, however, led to significantly lower riboswitch activities in a reporter system *in vivo* in *E. coli*. Importantly, when we substituted the amino- by azido-modified side chains, the cellular activities of the ligands were restored for the class-I conditional gene expression system and even improved for the class-II counterpart. Kinetic analysis of ligand binding *in vitro* revealed enhanced on-rates for amino-modified derivatives while they were attenuated for azido-modified variants. This shows that neither high affinities nor fast on-rates are necessarily translated into efficient cellular activities. Taken together, our comprehensive study interconnects *in vitro* kinetics and *in vitro* thermodynamics of RNA-ligand binding with the ligands’ *in vivo* performance and thereby encourages azido- rather than amino-functionalized design for enhanced cellular activity.

## Introduction

Riboswitches have emerged as possible targets for the development of alternative antimicrobial approaches [1–8]. They are typically located in the 5ʹ noncoding regions of bacterial mRNA and are able to bind specific metabolites to their aptamers with very high selectivity [9–11]. In a manner that is dependent on metabolite concentration, nascent mRNAs containing riboswitch domains can enter one of two mutually exclusive folding pathways to impart regulatory control [12]. The outcome of the folding pathway corresponds to ligand-bound or -free state. Thereby, the aptamer fold triggers structural cues into the expression platform which, in turn, transduces an ‘on’ or ‘off’ signal for gene expression, predominantly at the transcriptional or translational level [13–15].

One of the most critical steps in riboswitch gene regulation is ligand-sensing by the aptamer. For most riboswitches, the ligand becomes almost completely encapsulated by the RNA scaffold. Besides nucleobase stacking, most riboswitch aptamers involve every possible hydrogen donor or acceptor position of the ligand in hydrogen bond interactions with nucleotides of the binding pocket. This makes the structure-based design of modified ligand analogs and ligand mimics rather challenging. Nevertheless, the identification of novel potent ligands is a topic of intense research because ever since their discovery, riboswitches have been viewed as promising targets for the development of novel antibiotic strategies [16]. Likewise, efforts to engineer riboswitches for imaging purposes [17–19] or as biotechnological tools for the detection of endogenous and non-endogenous small molecules are in the focus of synthetic biologists interested in understanding and reprogramming cellular behavior [20].

In the present study, we examine the structure-activity relationships between 7-aminomethyl-7-deazaguanine (preQ_1_) sensing riboswitches [21] and chemically functionalized preQ_1_ ligands, both *in vitro* and *in vivo*. In particular, we ask the question how ‘add-on’ functionalities such as aminoalkyl, azidoalkyl, and ethylene glycol moieties that can potentially interact with the phosphate backbone and that are amenable for further derivatization, impact binding thermodynamics and kinetics and how the obtained *in vitro* parameters translate into riboswitch activity in the cell.

## Results and discussion

### Structure-based design of functionalized ligands for preQ_1_-I and -II riboswitches

PreQ_1_ is an intermediate of the biosynthesis pathway of the hypermodified nucleoside queuosine. Although queuosine is found in specific tRNAs of most eukaryotes and bacteria, it is only synthesized in bacteria. The queuosine modification enhances translational fidelity at the wobble position [22–25], and queuosine deficiency in bacteria can lead to reduced growth fitness and diminished virulence [26,27]. Bacterial riboswitches responsive to preQ_1_ are currently known to fall into three phylogenetically distinct classes. The preQ_1_-I (class 1) aptamer is distributed widely and rather compact, comprising not more than 34 nucleotides [28]. The preQ_1_-II (class 2) riboswitch is about twice this size and has been found in the *Firmicutes* [29]. Both classes are prevalent among important pathogens, such as *Streptococcaceae*. By contrast, the preQ_1_-III (class 3) riboswitch has been found exclusively in *Clostridium*, and it is the largest of all preQ_1_ riboswitches [21].

**Figure 1.**
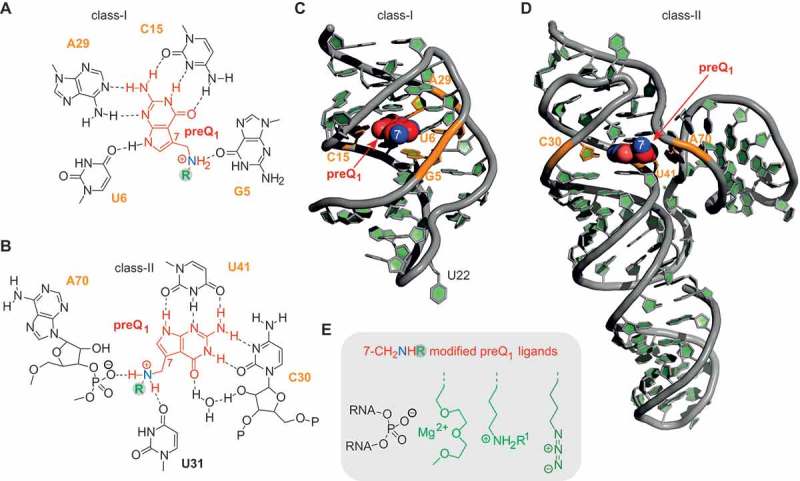
Structural analyses of preQ_1_ riboswitches. **A**) H-bond interactions between preQ_1_ and RNA binding pocket of class-I riboswitch; **B**) H-bond interactions between preQ_1_ and RNA binding pocket of class-II riboswitch; **C**) Three-dimensional structure of the *Thermoanaerobacter tengcongensis* (*Tte*) class-I preQ_1_ riboswitch (PDB ID: 3Q50); **D**) Three-dimensional structure of the *Lactobacillus rhamnosus* class-II preQ_1_ riboswitch (PDB ID: 4JF2); **E**) ‘Add-on’ functionalities of preQ_1_ ligands with the potential to interact with phosphate backbone units at the entrance of the ligand pocket to support ligand-RNA binding.

In the present comprehensive study, we have focused on the two most widespread classes of preQ_1_ riboswitches (I and II). These two classes employ distinct ligand binding modes (Figure 1) [30,31]. The class-I riboswitch recognizes preQ_1_ with cytosine (C15) through classical Watson–Crick base pairing and additionally through bidentate interaction of the ligand’s N3 and C2-NH_2_ with the *trans* Watson–Crick face of adenosine (A29) (Figure 1A,C). Moreover, the N9-H of preQ_1_ is H-bonding to the carbonyl O4 of uridine (U6) and the 7-aminomethyl moiety is involved in a further H-bond, namely to O6 of guanosine (G5).

The class-II riboswitch binds preQ_1_ differently (Figure 1B,D). PreQ_1_ pairs in bidentate fashion through *trans* Watson-Crick/Watson-Crick to cytidine (C30) and tridendate *via* N9-H–N3–C2-NH_2_ to the *trans* Watson–Crick face of uridine (U41). The O6 of the preQ_1_ lactam moiety forms a water-mediated bridge to the 2ʹ-OH of C30. Compared to the class-I riboswitch, the 7-aminomethyl group of the ligand is more strongly involved in interactions with the RNA, namely H-bonding to O6 of U31 and *via* electrostatic interactions to the phosphate group between A70 and A71.

Although preQ_1_ binding modes of class I and II riboswitches are different with respect to H-bonding patterns, for both riboswitches the 7-aminomethyl group of the ligand remains solvent-accessible in the bound state. The 7-aminomethyl group therefore appears to be a suitable anchor for tether attachment without disturbing ligand-aptamer recognition. Because we intended to retain the interaction characteristics of the 7-aminomethyl moiety, its alkylation (resulting in secondary amines) rather than acylation (resulting in amides) was considered to provide the most fitting functionality for attachments.

**Figure 2.**
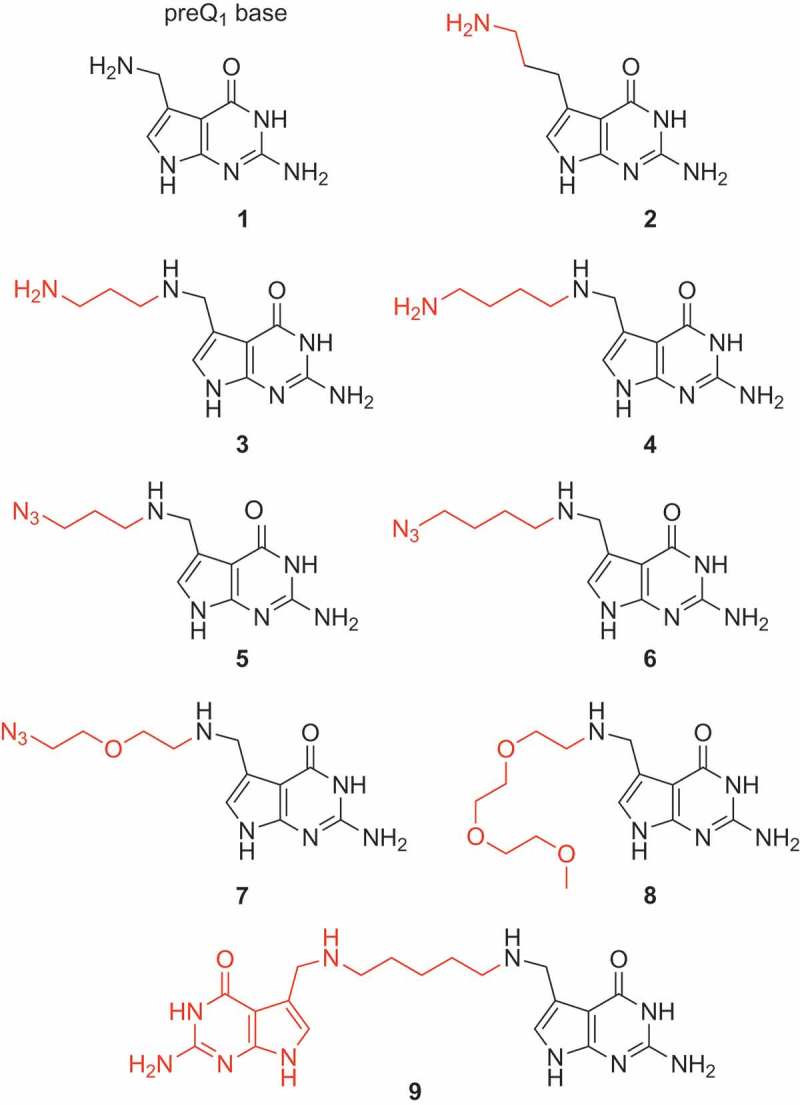
Chemical structures of the preQ_1_ ligand derivatives synthesized for this study (for synthetic details see the Supporting Information).

For tethering additional functionalities to the native ligand, we have mainly focused on two types of modifications. *First*, aminoalkyl tethers as shown for derivative **3** and **4** (Figure 1E, Figure 2) in their protonated form at suitable pH should be able to support binding of the modified ligand, based on specific electrostatic interactions with the phosphate backbone at the entrance of the ligand binding site. Higher affinities can be expected and additionally, binding kinetics are likely influenced due to an apparent increase in concentration because of non-specific interactions of the ammonium groups with the RNA phosphate backbone.

*Second*, azidoalkyl tethers as shown for derivative **5, 6** and **7** (Figure 1E, Figure 2) have been envisaged because of their potential for further straightforward functionalization with labeling compounds, e.g. fluorophores or biotin, using bioorthogonal Click or Staudinger reactions.

Moreover, we set out to analyze the impact of ethylene glycol moieties as shown for compound **8** (Figure 1E, Figure 2). In a different context, earlier studies on oligoribonucleotide duplexes carrying this functionalization demonstrated a Mg^2+^-chelating effect that can be utilized for enthalpic stabilization of RNA double helices [32]. We therefore speculated that this effect might be advantageous to stabilize small molecule–RNA interactions as well. Finally, we wanted to analyze the binding properties of a ligand dimer **9** (Figure 2) that bridges two preQ_1_ units via a short pentane linker to both 7-aminomethyl groups.

### Synthesis of tethered preQ_1_ ligands

To get access to the preQ_1_ derivatives displayed in Figure 2, we have developed a robust protocol for reductive amination of 7-(aminomethyl)-7-deazaguanine **1** [33] and the corresponding phthalimido-protected aminoalkylaldehydes and azidoalkylaldehydes, respectively, using tetramethylammonium triacetoxyborohydride in dimethylformamide and acetic acid. The phthalimido group was then cleaved with aqueous hydrazine solution. All tethered preQ_1_ derivatives were purified by reversed-phase chromatography applying an acetonitrile gradient to aqueous eluents containing one percent of trifluoroacetic acid. The products were thus obtained as salts of trifluoroacetic acid in excellent purity. Details of preparation are given in the Supporting Information. The developed routes provide significantly higher yields compared to direct alkylation of preQ_1_ using bromoalkyl substrates [34].

### Binding thermodynamics and kinetics of tethered preQ_1_ ligands to class-I and -II riboswitches

**Figure 3.**
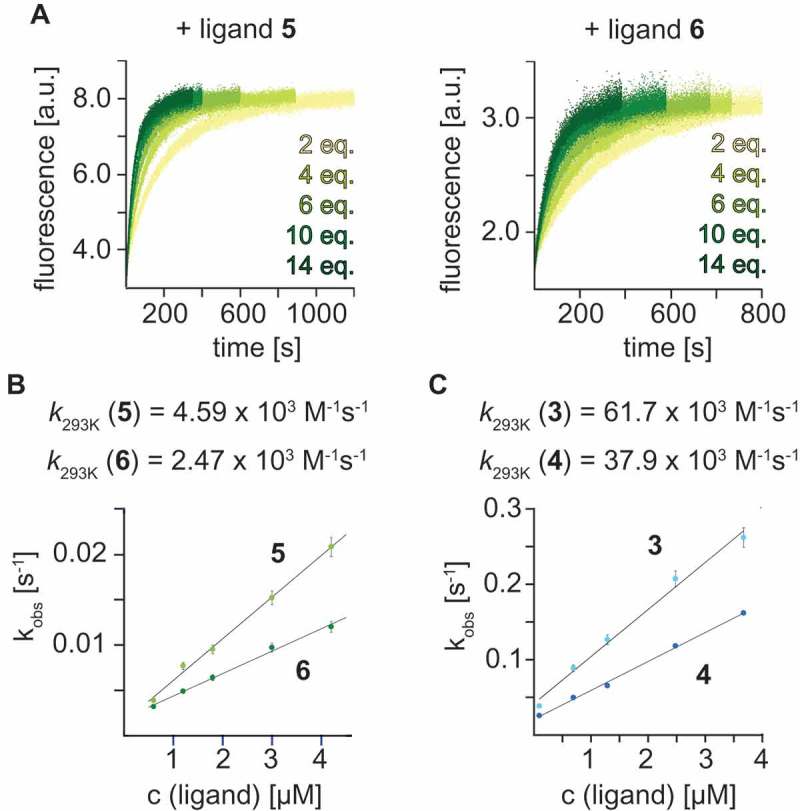
Stopped-flow fluorescence spectroscopy was used to monitor the kinetics of ligand preQ_1_ binding to the *Tte* preQ_1_ class-I riboswitch. **A**) Real time aminopurine (Ap) fluorescence time traces of the *Tte* U22Ap variant at different concentrations of 3-azidopropyl- and 4-azidobutyl-modified preQ_1_ **5** and **6; B**) Rate constants *k*_293_ from plots of observed rate *k*_obs_ versus ligand concentration. **C**) Same as (**B**) but for the 3-aminopropyl and 4-aminobutyl-modified preQ_1_ **3** and **4**. c(RNA) = 0.3 µM, c(MgCl_2_) = 2 mM, 100 mM KCl, 50 mM MOPS, pH 7.5, 293 K. Ligand concentration c(ligand) as indicated.

**Figure 4.**
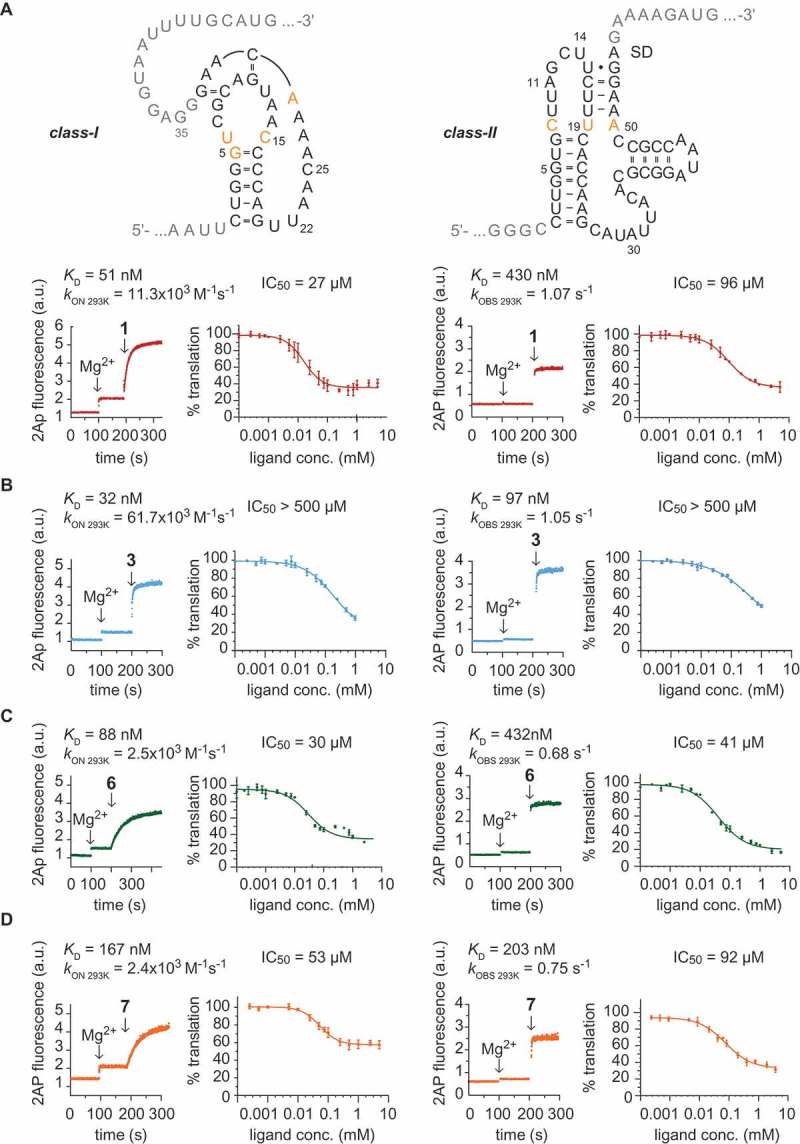
Comparison of *in vitro* and *in vivo* performance of preQ_1_ class-I and -II riboswitches with preQ_1_ and selected derivatives. **A**) Sequences of the *Tte* class-I (top left) and *Streptococcus pneumoniae* (*Spn*) class-II (top right) riboswitches used in this study. Nucleobase letters in black indicate the synthetic RNA aptamer sequences for *K*_D_ and *k*_on_ determinations (U22Ap and A11Ap substitution, respectively). Nucleobase letters in grey indicate the integration of the aptamers into the reporter mRNA. Letters in orange indicate nucleobases of the binding pocket that directly interact with the ligand via H-bonding. Exemplary fluorescence time traces of Ap-labeled preQ_1_ RNAs in response to Mg^2+^and preQ_1_ ligand **1** (conditions: 0.5 μM RNA, 100 mM KCl, 50 mM MOPS, pH 7.5, 293 K. Ligands: 2 mM MgCl_2_, 5 μM preQ_1_ **1**); affinities *K*_D_ were obtained from plots of normalized AP fluorescence intensities plotted as a function of ligand concentrations (for details see Fig. S1); rate constants *k*_on(293)_ of the *Tte* preQ_1_ class-I riboswitch were obtained from plots of observed rates *k*_obs_ *vs* ligand concentrations (for details see Figure 3 and Fig. S3); binding to the *Spn* preQ_1_-II riboswitch was independent of ligand concentration; for details of *k*_obs_ determination see Fig. S4. Dose-dependent repression of gene expression: B105 *E. coli* cells transformed with constructs expressing eGFP under translational control of preQ_1_ class-I and -II riboswitches were assayed for eGFP fluorescence in the presence of different preQ_1_ **1** concentrations (0.10 μM to 5 mM). Data represent the mean of three biological replicates, with error bars indicating standard deviation. The data were fit with a four-parameter logistic function to derive the IC_50_ values as indicated. **B**) Same as (**A**), but for ligand derivative **3. C**) Same as (**A**), but for ligand derivative **6. D**) Same as (**A**), but for ligand derivative **7.**

Ligand affinities (*K*_D_) as well as on-rates (*k*_on_) for ligand binding were measured based on a fluorescence spectroscopic approach (Fig. S1, Figure 3) that utilizes site-specifically 2-aminopurine (Ap) labeled RNA [35]. For preQ_1_ class-I riboswitches, we focused on the specific aptamer sequence from *Thermoanaerobacter tengcongensis (Tte)*; for preQ_1_ class-II riboswitches, we used the aptamer sequence from *Streptococcus pneumoniae (Spn)* (Figure 4A). For both, suitable positions for Ap substitutions (U22Ap class I; A11Ap class II) have been identified [36–38].

Of note, the availability of the 7-aminomethyl group of native ligand **1** is particularly important for class-II riboswitches. Affinity was 7-fold reduced for ligand analog **2** (7-(3-aminopropyl)-7-deazaguanine; Fig. S2A) that comprised an alkyl spacer placing the amino group at greater distance from the ligand core (Figure 2); likely, the longer chain hinders positioning of the amino group to generate contacts to the phosphate of A70–A71 and to U31 in the RNA binding pocket.

Concerning the class-I preQ_1_ riboswitch aptamer, the *K*_D_ values of amino- and azidopropyl and -butyl tethered ligands (**3** to **6**) measured in aqueous buffer at pH 7.5 (50 mM MOPS, 100 mM KCl, 293 K) in the presence of 2 mM MgCl_2_ were comparable to native ligand **1**, varying only by a factor of 1.7 (Table 1, Figure 4B-D, Fig. S1 and S2). Ligands carrying longer chains, such as azidoethoxyethyl-preQ_1_ **7** and triethylene glycol-linked preQ_1_ **8**, experienced a 4-fold and 18-fold decrease in affinity, respectively (Table 1, Fig. S2). We speculate that the conformational changes of the tethered group that are required for a chelating Mg^2+^ interaction (as indicated in Figure 1E) might lead to an entropic destabilization that compensates the enthalpic stabilization. Also, for the preQ_1_ dimer **9**, a loss in affinity (11-fold) was found (Table 1).

**Table 1.** Class-I preQ_1_ riboswitches – Thermodynamic and kinetic parameters of ligand binding and cellular activity.

Ligand No.	*K*_D_ [nM]	*k*_on_ [M^−1^s^−1^ x10^−3^	*k*_off_ [s^−1^] *	IC_50_ [µM] **
1	51	11.3	0.000576	27 (75%)
2	71	4.3	0.000305	n.d.
3	32	61.7	0.001974	> 500
4	41	37.9	0.001554	> 1000
5	30	4.6	0.000138	52 (65%)
6	88	2.5	0.000220	30 (70%)
7	167	2.4	0.000401	53 (42%)
8	922	3.4	0.003135	152 (56%)
9	590	3.5	0.002065	308 (38%)

* off-rates were calculated from k_off_ = K_D_ x k_on_ (ref. [51]);

** number in brackets represents percentage of translational repression at saturating ligand concentration;

n.d. not detectable

Interestingly, for the preQ_1_ class-II riboswitch aptamer, the affinities measured for aminopropyl and -butyl tethered ligands (**3** and **4**) were increased by 3- to 4-fold compared to the native ligand, consistent with stabilizing interactions between the additional ammonium group and the RNA phosphate backbone at the entrance of the binding pocket (Table 2, Figure 4B, Fig. S1B and S2A). In contrast to **3** and **4**, the *K*_D_ values of the corresponding azidopropyl and -butyl tethered ligands (**5** and **6**) were comparable to the native ligand, varying only by a factor of 1.7 (Table 2, Figure 4C, Fig. S1B and S2C). Also, for azidoethoxyethyl-preQ_1_ **7** the affinity remained comparable, however, the triethylene glycol-linked preQ_1_ **8** and the preQ_1_ dimer **9**, experienced a 10-fold and 4-fold loss in affinity, respectively (Table 2). From this *in vitro* analysis, it becomes obvious that varying affinities of a particular ligand derivative towards class-I and class-II aptamers likely originate from their distinct structural features leading to differential accomodation and interaction with the tether.

**Table 2.** Class-II preQ_1_ riboswitches – Thermodynamic and kinetic parameters of ligand binding and cellular activity.

Ligand No.	*K*_D_ [nM]	*k*_obs_ [s^−1^]	*k*_off_ [s^−^[^1]^] *	IC_50_ [µM] **
1	430	1.07	0.084733	96 (65%)
2	2830	> 10***	n.d.	n.d.
3	97	1.05	0.019982	> 500
4	148	0.80	0.022999	> 1000
5	254	0.70	0.033841	71 (80%)
6	432	0.68	0.054080	40 (80%)
7	203	0.75	0.029262	92 (68%)
8	4580	0.72	0.031532	116 (65%)
9	1030	1.06	0.181061	328 (53%)

* off-rates were calculated from *k*_off_ = *k*_obs_/(1+([L]/*K*_D_)) (ref. [51]);

** number in brackets represents percentage of repression at saturating ligand concentration;

*** estimated value;

n.d. not determined

With regard to ligand binding kinetics, we note that previous studies have demonstrated that for the *Tte* class-I aptamer, preQ_1_ binding kinetics are strongly dependent on preQ_1_ concentrations [36]. Employing the U22Ap riboswitch variant for the 2ApFold fluorescence approach here, we determined an on-rate *k*_on_ of 11.3 × 10^3^ M^−1^s^−1^ for preQ_1_ **1** (Table 1). For the *Spn* class-II preQ_1_ counterpart, however, we found that binding kinetics of preQ_1_ **1** (based on the corresponding A11Ap variant) were independent of ligand concentration, with *k*_obs_ of 1.07 ± 0.30 s^−1^ (over the same range of 2 to 14-fold excess of ligand over RNA as applied to the class-I counterpart). This suggests that a conformational change or conformational adaption of the class-II preQ_1_ RNA is possibly rate-limiting for the ligand binding process.

For the aminoalkyl ligand derivatives **3** and **4** we found three to five-fold faster on-rates *k*_on_ for binding to the class-I aptamer compared to native preQ_1_ **1** (Table 1, Figure 3C, Fig. S2B and S3). By contrast, the corresponding azidoalkyl ligands **5** and **6** with the same tether lengths were observed to have two and five-fold slower on-rates *k*_on_ than preQ_1_ **1** for binding to the class-I aptamer (Table 1, Figure 3B, Fig. S2C). The faster on-rates for ligands **3** and **4** are consistent with the possibility for specific electrostatic interactions between the additional ammonium moieties and the phosphate backbone, and more generally, with an increase in local concentration due to improved electrostatic interactions with the negatively charged RNA. They are also consistent with an earlier proposed induced-fit binding mode of the preQ_1_ class-I riboswitch [39,40].

Not unexpectedly, for the class-II riboswitch where ligand binding is not the rate limiting step but likely a conformational change that occurs in the RNA pocket, all preQ_1_ ligand derivatives **3** to **9** exhibited rates *k*_obs_ that were comparable to that of the native ligand (Table 2, Figure 4, Fig. S2 and S4). Only a slight rate difference among the ligand derivatives was observed, with azidobutyl modified preQ_1_ **6** being slowest (*k*_obs_ 0.68 ± 0.30 s^−1^) and aminopropyl modified preQ_1_ **3** together with dimer **9** being fastest (*k*_obs_ 1.05 ± 0.16 s^−1^ and *k*_obs_ 1.06 ± 0.11 s^−1^) (Table 2, Fig. S4). The here observed concentration-independence of class-II riboswitches with respect to binding rates is consistent with the conformational capture model that was deduced from NMR spectroscopic investigations [41]. This model proposed that stem P4 is poised to act as a ‘screw cap’ on preQ_1_ recognition to block ligand exit and stabilize the binding pocket.

### Cellular activity of functionalized preQ_1_ derivatives in a preQ_1_ deficient E. coli strain

Previously, preQ_1_ riboswitches have attracted attention as platforms for the engineering of orthogonal riboswitches to control gene expression. *Micklefield* and coworkers used a rational targeted approach in the evaluation of synthetic compounds with riboswitch mutants and identified an orthogonal riboswitch−ligand pair that effectively repressed the transcription of selected genes in *B. subtilis* [42]. More recently, rationally engineered preQ_1_ riboswitches have been applied for inducible gene regulation in mycobacteria [43].

In this study, we investigated how the affinity and kinetic parameters obtained *in vitro* for functionalized preQ_1_ derivatives (**2** to **9**) translate into cellular activity (Figure 4). We therefore engineered a preQ_1_ class I or class II riboswitch- controlled reporter gene (green fluorescence protein, GFP) and monitored its production in response to the different ligands *in vivo* in *E. coli*. To avoid potential interference of endogenous preQ_1_ with the assay, we used an *E. coli* strain bearing an inactivating mutation of the *queC* gene, which encodes a protein involved in the early steps of queuosine synthesis [44]. *Tte* and *Spn* preQ_1_ riboswitches act at the level of translation by sequestering the Shine-Dalgarno sequence via ligand-triggered alternative RNA folding [21]. Successful binding of the preQ_1_ ligand therefore results in a decrease of GFP production, which can be measured directly in the bacterial culture by determining GFP fluorescence. We used an inducible reporter system (pQE70 bacterial expression system) to repress GFP transcription in the absence of the inducer (IPTG) and added the different ligands concomitantly with the IPTG. Fluorescence measurements at 6 h after induction revealed that the native preQ_1_ ligand **1** was capable of dose-dependent regulation of class I–controlled GFP expression with an IC_50_ value of 27 μM and 75% repression observed at preQ_1_ concentrations of 1 mM or higher (Figure 4A) while for the class II riboswitch, the IC_50_ value amounted to 96 μM with 65% repression at 1 mM or higher preQ_1_ concentrations (Figure 4A).

We then evaluated the importance for *in vivo* activity of the native 7-aminomethyl group as a structural subunit in analogs of preQ_1_ by measuring IC_50_ values of compound **2**. Although this compound comprises the 7-deazaguanine core, the replacement of the 7-aminomethyl by a 7-(3-aminopropyl) substituent renders it practically inactive *in vivo* regardless of the riboswitch tested (Fig. S2A). This was especially surprising in the case of the class I riboswitch because *in vitro*, compound **2** displayed nearly the same affinity to class I aptamers as the native ligand.

Unexpectedly, the aminoalkylated ligands **3** and **4** that showed up to 4-fold higher affinities (class-II) and up to 5-fold increased on-rates (class-I) *in vitro* exhibited poor regulation ability *in vivo*. IC_50_ values for both riboswitch classes were at least 10-fold higher than those for native preQ_1_ **1** (Table 1, Figure 4B, Fig. S2B). However, when azido instead of amino groups were present at tethers of the same lengths, as in preQ_1_ derivatives **5** and **6**, the *in vivo* activity was restored (for class I) or even improved (for class II) compared to the native preQ_1_ **1** ligand (Table 1, Figure 4C, Fig. S2C). Intriguingly, those ligands had shown significantly slower on-rates in *in vitro* binding studies (for class-I) (Figure 4C). Together, these findings demonstrate that azidoalkylated ligands exhibit excellent bioavailability and are potent triggers of riboswitch conformation changes resulting in the repression of translation *in vivo*. On the other hand, it appears that despite superior *in vitro* affinity of aminoalkylated ligands, they are less suitable for *in vivo* applications. Potential reasons for that could be reduced cellular uptake [45] or interference with polyamine metabolism in the cell [46].

We also tested the *in vivo* activity of azidoethoxyethyl preQ_1_ derivative **7** and found that it was comparable to that of the native preQ_1_ ligand for the class II riboswitch and slightly higher for the class I type (Figure 4D). The triethylene glycol modification of the ligand (derivative **8**) resulted in strongly decreased *in vitro* affinities of this ligand for both riboswitch classes. Interestingly, however, the *in vivo* activity of derivative **8** towards the class-II riboswitch was essentially equal to the native ligand (Fig. S2D). For the class-I riboswitch, a similar trend was observed in that the decrease in IC_50_ value was less pronounced than expected considering its low affinity *in vitro* (Fig. S1D). Finally, the ligand dimer **9** showed clearly inferior *in vivo* activity towards both class-I and -II riboswitches compared to the native ligand **1** (Tables 1 and 2).

## Conclusions

For several reasons riboswitches have been considered attractive targets for antimicrobial drug development [1–5]: They are well structured and allow stable binding of low–molecular weight compounds to RNA with affinities as found for interactions between established antibiotics and ribosomal RNA. Furthermore, riboswitches have not been identified in mammals which should reduce the risk of undesired side effects. Finally, they are often located upstream of genes encoding enzymes that are involved in the synthesis of the metabolite that triggers the very riboswitch. By designing suitable metabolite analogs that outcompete the natural ligand for interaction with the riboswitch, the production of the metabolite will be inhibited by preventing the expression of the synthesis genes. If the respective metabolite is essential for life, this will lead to a growth stop and/or death of the bacterial cell. Several studies have demonstrated that riboswitches are indeed druggable [6–8,16,47–49]. The most prominent investigation employed a phenotypic screen and identified ribocil that acts as a structurally distinct mimic of the natural ligand flavin mononucleotide to repress *ribB* gene expression and inhibit cell growth [7].

Modest success, however, derived from ligand design that relied on the modulation of the nature and/or position of heteroatoms of the ligand core and/or the decoration of accessible positions with substituents that are typically used in medicinal chemistry as e.g. reported recently for guanine-sensing riboswitches in the bacterial pathogen *Clostridioides difficile* [6]. To the best of our knowledge, such studies have not yet included the evaluation of azide functionalization of ligands. Our study now demonstrates that the attachment of short azido-tethers to the native ligand of preQ_1_ riboswitches leads to improved efficacy (> 2-fold decreased IC_50_) and significantly increased repression (from 65% to 80%) of a GFP reporter in *E. coli*. These findings were unexpected because the thermodynamic and kinetic parameters *k*_on_ and *K*_D_ determined *in vitro* were clearly inferior to amino-modified derivatives and rather similar to the native ligand. It was furthermore unexpected that the amino-modified derivatives that gave the highest affinities *in vitro*, exhibited the lowest cellular activities of all preQ_1_ derivatives investigated.

For future prospects, azido-modified preQ_1_ ligands due to their excellent bioavailability and *in vivo* activity may constitute highly promising platforms for *in vivo* labeling approaches. The here presented azido-tethered preQ_1_ derivatives are amenable for bioorthogonal labeling reactions with diverse reporter groups such as fluorophores. Recently, the structure-guided design of fluorescent *S*-adenosyl-L-methionine (SAM) analogs has been successfully introduced for a high throughput screen to target SAM-I riboswitch RNAs [50]. Such screens are likely expandable to preQ_1_ riboswitches based on the derivatives presented. Finally, inspiration for live cell imaging applications of preQ_1_-fluorophor conjugates can be drawn from a recent RNA imaging assay using the cobalamin riboswitch as an RNA tag and a series of probes containing the cobalamin ligand as a fluorescence quencher to elicit fluorescence turn-on upon binding RNA [17].
