# Supplementary material for: Superior cellular activities of azido- over amino-functionalized ligands for engineered preQ1 riboswitches in E.coli
Source: RNA Biol. 2018 Oct 26;15(10):1376–83. doi: 10.1080/15476286.2018.1534526 (PMC6284575; doi:10.1080/15476286.2018.1534526)
Supplement: Supplemental Material [file krnb-15-10-1534526-s001.pdf]

Supporting Information

Superior cellular activities of azido- over amino-functionalized ligands for engineered preQ$_1$ riboswitches in *E. coli*

Eva Neuner, Marina Frener, Alexandra Lusser, Ronald Micura

Contents

I. Supporting Methods

1. Synthesis of compound **2** 2
2. Synthesis of compound **3** 6
3. Synthesis of compound **4** 8
4. Synthesis of compound **5** 9
5. Synthesis of compound **6** 11
6. Synthesis of compound **7** 15
7. Synthesis of compound **8** 17
8. Synthesis of compound **9** 19
9. Solid-phase synthesis of oligonucleotides 20
10. Deprotection of oligonucleotides 20
11. Purification of RNA 20
12. Mass spectroscopy of RNA 21
13. Determination of ligand binding affinities (K_D) 21
14. Determination of ligand binding kinetics (k_{obs} and k_{on}) 22
15. Cellular assays (IC_{50}) 22
16. References 23

II. Supporting Figures

Supporting Figure **S1** 25
Supporting Figure **S2** 26
Supporting Figure **S3** 27
Supporting Figure **S4** 28

1. Synthesis of compound 2

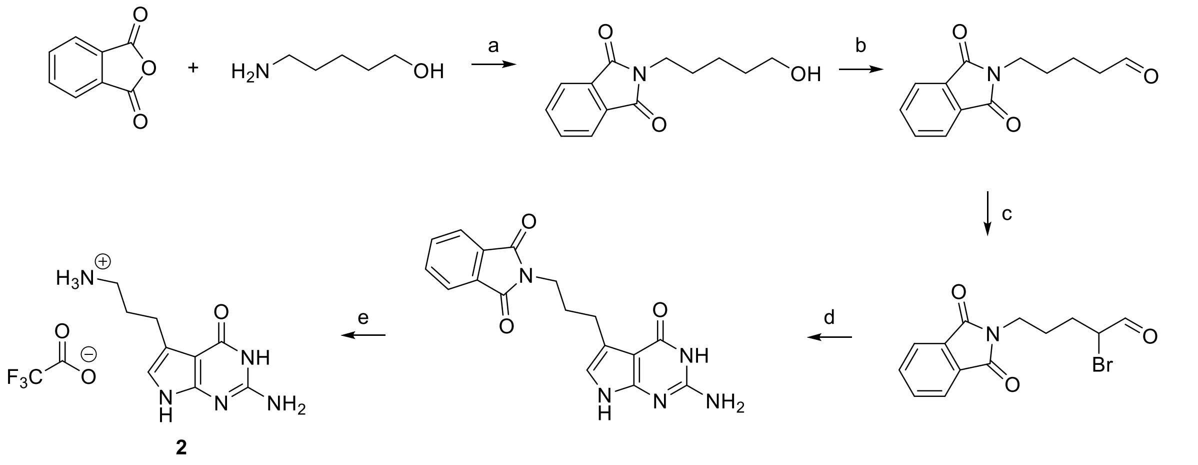

Scheme 1: Synthesis of compound **2**. a) Et_3N, toluene, reflux, 4 h, 81%. b) TCCA (1 eq), TEMPO, CH_2Cl_2, RT, 30 min., 58%. c) DBBA (0.6 eq), ACN, reflux, 4 h, 73%. d) 2,4-diaminopyrimidinone (1 eq), NaOAc (2 eq), H_2O/ACN, 40°C, 4 h, 58%. e) H_2NNH_2 (10 eq), EtOH, reflux, overnight, TFA (1%), RT, overnight, 83%.

1.1. *N*-(5-Hydroxypentyl)phthalimide

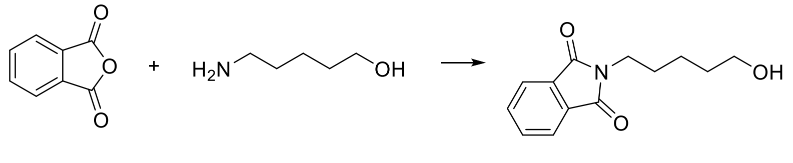

Aminopentanol (2.820 g, 27.3 mmol) was dissolved in tolune (41 ml) and phthalic anhydride (4.049 g, 27.3 mmol) and triethylamine (273 mg, 2.7 mmol) were added. The reaction mixture was heated to 130°C and refluxed for 4 hours. The solvents were evaporated, and the oily residue was dissolved in ethyl acetate. The organic phase was washed with 1 M HCl, saturated $NaHCO_3$ and saturated NaCl, dried over Na_2SO_4 and evaporated.

Yield: 5.158 g (81%) colorless oil

TLC (ethylacetate/cyclohexane, 1:1): R_f = 0.32

^{1}H-NMR (300 MHz, $CDCl_3$): δ 1.39 – 1.46 (m, 2H, H_2-C(3)); 1.57 – 1.76 (m, 4H, H_2-C(2), H_2-C(4)); 3.61 – 3.72 (m, 4H, H_2-C(1), H_2-C(5)); 7.71 (d, 2H, H-C(aromat.)); 7.83 (d, 2H, H-C(aromat.)) ppm.

1.2. *N*-(5-Oxopentyl)phthalimide

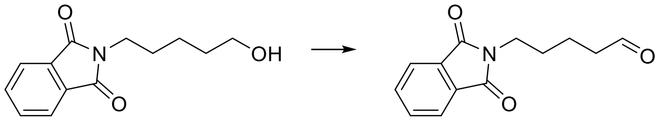

5-Hydroxypentylphthalimide (1.195 g, 5.12 mmol) was dissolved in dry dichloromethane (15 ml) and cooled to 0°C in an ice bath. Trichloroisocyanuric acid (1.190 g, 5.12 mmol) and 2,2,6,6-tetramethylpiperidinyloxyl (TEMPO) (8 mg, 0.0512 mmol) were added and a yellow suspension was formed. The reaction mixture was stirred at room temperature for 30 minutes and was then filtered through a celite pad. The organic phase was washed with saturated $NaHCO_3$, 1 M HCl and saturated NaCl, dried over Na_2SO_4, evaporated and dried under high vacuum.

Yield: 682 mg (58%) yellow oil

TLC (ethylacetate/cyclohexane, 1:1): R_f = 0.5

^{1}H NMR (300 MHz, $CDCl_3$): δ 1.66 – 1.76 (m, 4H, H_2-C(2), H_2-C(3)); 2.50 (t, 2H, H_2-C(4)); 3.71 (t, 2H, H_2-C(1)); 7.70 – 7.73 (m, 2H, H-C(arom.)); 7.83 – 7.86 (m, 2H, H-C(arom.)); 9.76 (s, 1H, HCO) ppm.

1.3. *N*-(4-Bromo-5-oxopentyl)phthalimide

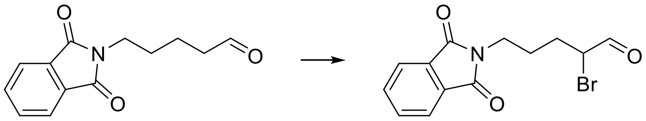

N-(5-oxopentyl)phthalimide (400 mg, 1.73 mmol) was dissolved in acetonitrile (5 ml) and 5,5-dibromobarbituric acid (297 mg, 1.03 mmol) was added. The solution was refluxed for 4 hours, cooled to room temperature and evaporated. The crude product was purified by column chromatography on silica gel (ethylacetate/cyclohexane, 2:8 – 3:7).

Yield: 392.6 mg (73%) colorless oil

TLC (ethylacetate/cyclohexane, 2:1): R_f = 0.72

^{1}H NMR (400 MHz, $CDCl_3$): δ 2.05 – 2.11 (m, 2H, H_2-C(2)); 2.46 – 2.50 (m, 2H, H_2-C(3)); 3.72 (t, 1H, H-C(4)); 3.78 (t, 2H, H_2-C(1)); 7.69 – 7.72 (m, 2H, H-C(arom.)); 7.81 – 7.84 (m, 2H, H-C(arom.)); 9.17 (1H, H-CO(5)) ppm.

1.4. 7-(3-Phthalimidopropyl)-7-deazaguanine

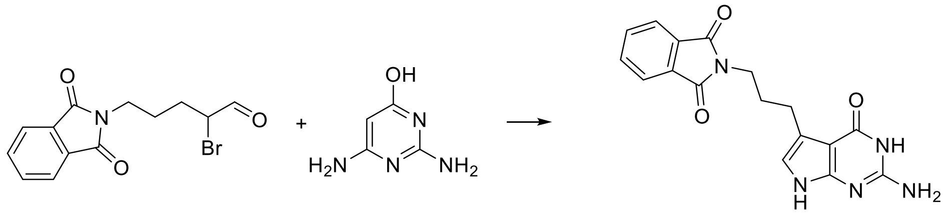

N-(4-Bromo-5-oxopentyl)phthalimide (390 mg, 1.6 mmol) was dissolved in water/acetonitrile (1/1) (5 ml) and 2,4-diaminopyrimidinone (159 mg, 1.26 mmol) and sodium acetate (207 mg, 2.52 mmol) were added. The reaction mixture was stirred at 40°C for 4 hours and the product started to precipitate after 40 minutes. The product was filtered off and dried under high vacuum.

Yield: 246 mg (58%) orange solid

TLC (ethylacetate/cyclohexane, 1:1): $R_f = 0$

^{1}H NMR (400 MHz, d$_6$-DMSO): δ 1.91 (t, 2H,); 2.58 (t, 2H,); 3.58 (t, 2H, H_2-C(1′)); 5.96 (2H, H_2-N(2)); 6.38 (1H, H-C(8)); 7.81-7.87 (m, 4H, H-C(arom.)); 10.09 (1H, H-N(1)); 10.62 (1H, H-N(9)) ppm.

1.5. 7-(3-Aminopropyl)-7-deazaguanine trifluoroacetate 2

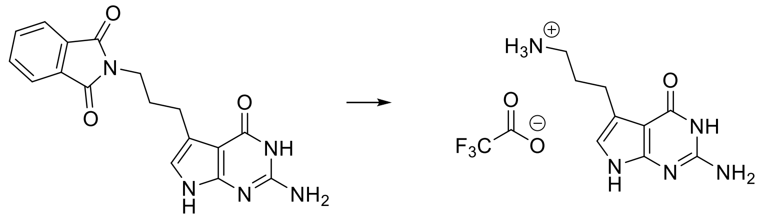

2

7-(3-Phthalimidopropyl)-7-deazaguanine (230 mg, 0.68 mmol) was dissolved in ethanol and hydrazine monohydrate (64%, 341 mg, 6.8 mmol) was added dropwise. The reaction mixture was refluxed overnight, the solvents were evaporated, and the crude product was dried under high vacuum. It was then suspended in trifluoroacetic acid (1%, 6 ml) and was shaken overnight at room temperature. The insoluble residue was filtered off and the filtrate was concentrated in vacuo. The crude product was purified by reversed phase chromatography on an ÄKTA prime plus system (LiChroprep® RP-18 (40 – 63 µm), 1% trifluoroacetic acid/acetonitrile, 100:0 – 70:30, 360 ml, flow rate 4 ml/min).

Yield: 182 mg (83%) of **2** as beige solid

HR-ESI-MS (m/z): $[M+H]^+$ calculated: [208.1193]; found: [208.1191]

^{1}H NMR (400 MHz, d_6-DMSO): δ 1.86 (2H, H_2-C(2′)); 2.63 (2H, H_2-C(1′)); 2.74 (2H, H_2-C(3′)); 6.19 (2H, H_2-N(2)); 6.43 (1H, H-C(8)); 7.68 (3H, H_3-N^+(3′)); 10.39 (1H, H-N(1)); 10.81 (1H, H-N(9)) ppm.

2. Synthesis of compound 3

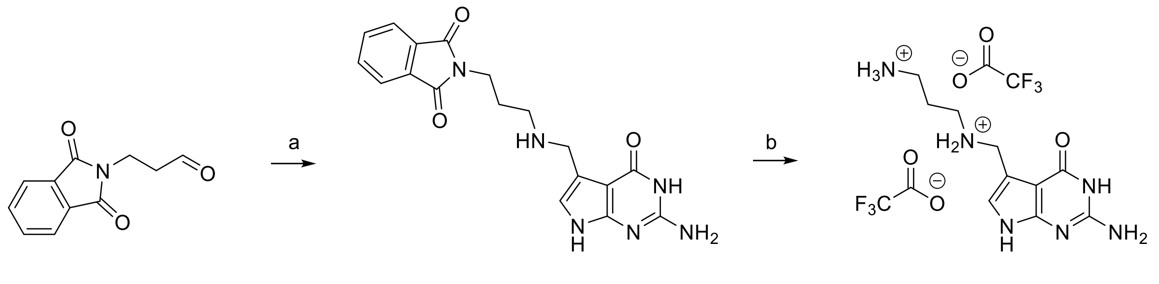

3

Scheme 2: Synthesis of compound **3**. a) preQ$_1$ (1.2 eq), $(CH_3)_4NHB(OAc)_3$ (2.5 eq), HOAc, DMF, RT, 5 h, 96%. b) H_2NNH_2 (10 eq), EtOH, reflux, overnight, TFA (1%), RT, overnight, 77%.

2.1. 7-((Phthalimidopropyl)aminomethyl)-7-deazaguanine

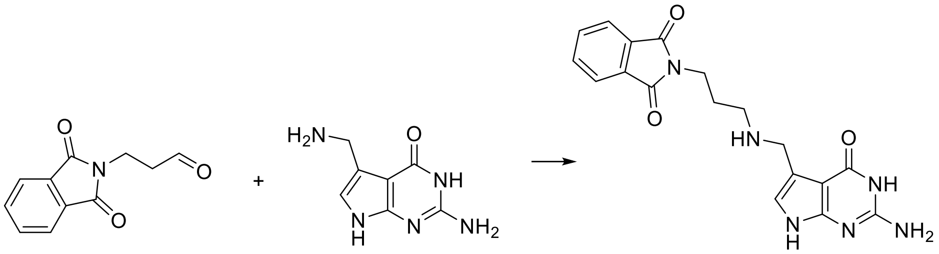

PreQ$_1$ (387 mg, 1.32 mmol) was dissolved in dimethylformamide (15 ml) and *N*-(3-oxopropyl)phthalimide (215 mg, 1.06 mmol) was added. After the successive addition of tetramethylammonium triacetoxyborohydride (697 mg, 2.65 mmol) and acetic acid (60 µl) the solution was stirred under argon at room temperature for 5 hours. The reaction mixture was concentrated in vacuo and the crude product was purified by column chromatography on silica gel (dichloromethane/methanol, 95:5 – 88:12).

Yield: 375 mg (96%) beige solid

TLC (dichloromethane/methanol, 85:15): R_f = 0.33

ESI-MS (m/z): $[M+H]^+$ calculated: [367.37], found: [367.15]

^{1}H NMR (300 MHz, d$_6$-DMSO): δ 1.98 (m, 2H, H_2-C); 2.99 (m, 2H, H_2-C); 3,65 (t, 2H, H_2-C); 4.13 (m, 2H, H_2-C(7)); 6.54 (s, 2H, H_2-N(2)); 6.80 (s, 1H, H-C(8)); 7.86 (d, 4H, H-C(arom.)); 9.23 (s, 1H, H-N(7)); 11.03 (s, 1H, H-N(1)); 11.27 (s, 1H, H-N(9)) ppm.

2.2. 7-((3-Aminopropyl)aminomethyl)-7-deazaguanine trifluoroacetate 3

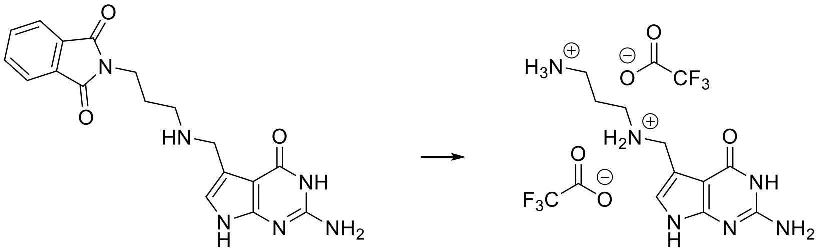

3

7-((Phthalimidopropyl)aminomethyl)-7-deazaguanine (375 mg, 1.02 mmol) was dissolved in ethanol (16 ml). Hydrazine monohydrate (64 %, 0.497 ml, 10.2 mmol) was added and the solution was refluxed overnight. The reaction mixture was concentrated in vacuo and the crude product was suspended in trifluoroacetic acid (1%, 20 ml) and shaken overnight at room temperature. The suspension was filtered off and the filtrate was concentrated in vacuo. The product was purified by reversed phase chromatography on an ÄKTA prime plus system (LiChroprep® RP-18 (40 – 63 µm), 1% trifluoroacetic acid/acetonitrile, 100:0 – 90:10, 360 ml, flow rate 4 ml/min).

Yield: 362 mg (77%) of **3** as beige solid

ESI-MS (m/z): $[M+H]^+$ calculated: [237.27], found: [237.29]

^{1}H NMR (300 MHz, d_6-DMSO): δ 1.91 (m, 2H, $H_2C(2')$); 2.87 (m, 2H, $H_2C(3')$); 3.01 (m, 2H, $H_2C(1')$); 4.17 (m, 2H, $H_2C(7)$); 6.40 (s, 2H, $H_2N(2)$); 6.83 (s, 1H, H-C(8)); 7.77 (m, 3H, ^+H_3N-C); 9.06 (m, 2H, ^+H_2N-C); 10.9 (s, 1H, H-N(9)); 11.3 (s, 1H, H-N(1)) ppm

3. Synthesis of compound 4

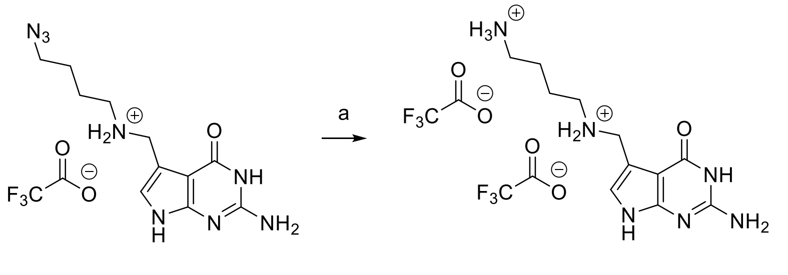

Scheme 3: Synthesis of compound **4**. a) Pd/C (10wt%), H_2, MeOH, RT, 3.5 h, 63%.

3.1. 7-((4-Aminobutyl)aminomethyl)-7-deazaguanine trifluoroacetate 4

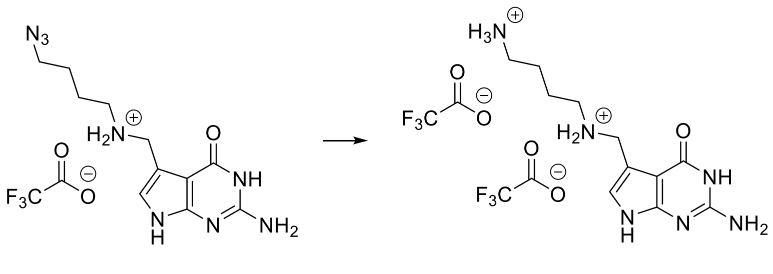

Azidobutyl-preQ$_1$ trifluroacetate (65 mg, 0.167 mmol) was dissolved in methanol (1.5 ml) and palladium on carbon (7.6 mg, loading 10wt%) was added. The suspension was stirred at room temperature and hydrogen in a balloon was bubbled through for half an hour. Afterwards the suspension was stirred under hydrogen atmosphere for 3 hours, diluted with methanol and filtered through a celite pad. The solution was concentrated in vacuo and the crude product was purified by reversed phase chromatography on an ÄKTA prime plus system (LiChroprep® RP-18 (40 – 63 µm), 1% trifluoroacetic acid/acetonitrile, 95:5, flow rate 3 ml/min).

Yield: 50 mg (63%) of **4** as beige solid

ESI-MS (m/z): $[M+H]^+$ calculated: [251.32], found: [251.03]

^{1}H NMR (300 MHz, d$_6$-DMSO): δ 1.59 (m, 4H, H_2-C(2',3')); 2.97 (m, 2H, H_2-C(4')); 2.80 (m, 2H, H_2-C(1')); 4.16 (m, 2H, H_2-C(7)); 6.42 (s, 2H, H_2-N(2)); 6.83 (s, 1H, H-C(8)); 7.74 (m, 3H, $^+H_3$N-C); 9.03 (m, 2H, $^+H_2$N-C); 10.9 (s, 1H, H-N(9)); 11.3 (s, 1H, H-N(1)) ppm.

4. Synthesis of compound 5

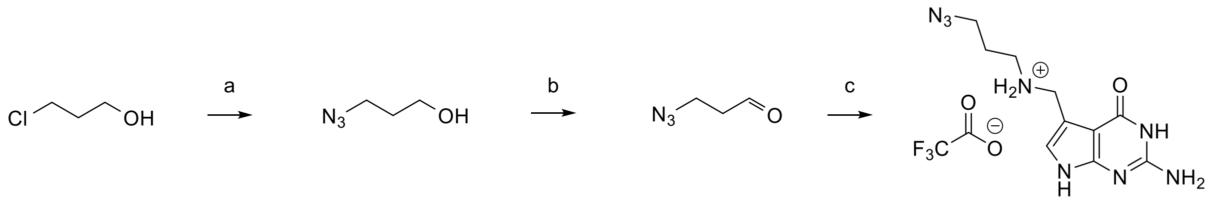

Scheme 4: Synthesis of compound **5**. a) NaN_3 (3 eq), DMF, 65°C, 22 h, 83%. b) Dess-Martin periodinane (1.3 eq), CH_2Cl_2, RT, 1 h, 92%. c) $preQ_1$ (1.1 eq), $(CH_3)_4NHB(OAc)_3$ (2 eq), HOAc, DMF, RT, 5 h, 47%.

4.1. 3-Azidopropanol

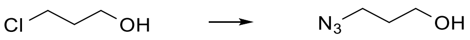

Sodium azide (4.68 g, 72 mmol) was suspended in dimethylformamide (16 m) and 3-chloropropanol (2 ml, 24 mmol) was added. The suspension was stirred under argon atmosphere for 22 hours at 65°C. After cooling to room temperature, the mixture was diluted with water, extracted with diethyl ether, washed with saturated $NaHCO_3$ and NaCl, dried over $MgSO_4$ and concentrated in vacuo.

Yield: 2.00 g (83%) colorless oil

TLC (pentane/diethyl ether, 75:25): R_f = 0.18

^{1}H NMR (300 MHz, d_6-DMSO): δ 1.66 (m, 2H, H_2-C(2)); 3.37 (m, 2H, H_2-C(1)); 3.46 (m, 2H, H_2-C(3));4.55 (t, 1H, OH) ppm.

4.2. 3-Azidopropanal

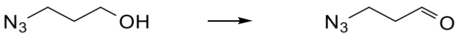

3-Azidopropanol (200 mg, 2.0 mmol) was dissolved in dry dichloromethane (20 ml) and Dess-Martin periodinane (1.10 g, 2.6 mmol) was added in portions. The reaction mixture was stirred for 1 hour at room temperature under argon atmosphere. Subsequently, the suspension was

filtered through a celite pad and the filtrate was concentrated in vacuo. The crude product was purified by column chromatography on silica gel (pentane/diethyl ether, 75:25).

Yield: 180 mg (92%) colorless oil

TLC (pentane/diethyl ether, 75:25): R_f = 0.21

^{1}H NMR (300 MHz, CDCl$_3$): δ 2.96 (t, 2H, H_2-C); 3.82 (t, 2H, H_2-C); 9.82 (d, 1H, H-CO) ppm.

4.3. 7-((3-Azidopropyl)aminomethyl)-7-deazaguanine trifluoroacetate 5

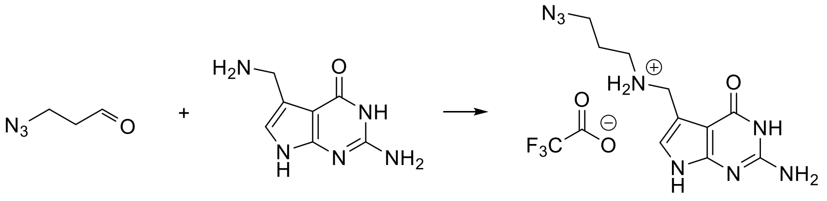

5

3-Azidopropanal (49 m, 0.49 mmol) was dissolved in dimethylformamide (10 ml) and preQ$_1$ trifluoroacetate (158 mg, 0.54 mmol) was added. After the addition of tetramethylammonium triacetoxyborohydride (258 mg, 0.98 mmol) and acetic acid (27 µl) the solution was stirred for 5 hours at room temperature. The reaction mixture was concentrated in vacuo and the crude product was purified by reversed phase chromatography on an ÄKTA prime plus system (LiChroprep® RP-18 (40 – 63 µm), 1% trifluoroacetic acid/acetonitrile, 100:0 – 70:30, 360 ml, flow rate 3 ml/min).

Yield: 87 mg (47%) of **5** as beige solid

ESI-MS (m/z): $[M+H]^+$ calculated: [263.28], found: [263.25]

^{1}H NMR (300 MHz, d$_6$-DMSO): δ 1.86 (m, 2H, H_2C(2')); 3.00 (m, 2H, H_2C(3')); 3.48 (m, 2H, H_2C(1')); 4.17 (m, 2H, H_2C(7)); 6.34 (s, 2H, H_2N(2)); 6.82 (s, 1H, H-C(8)); 8.94 (m, 2H, $^+H_2$N-C(7)); 10.9 (s, 1H, H-N(9)); 11.3 (s, 1H, H-N(1)) ppm.

5. Synthesis of compound 6

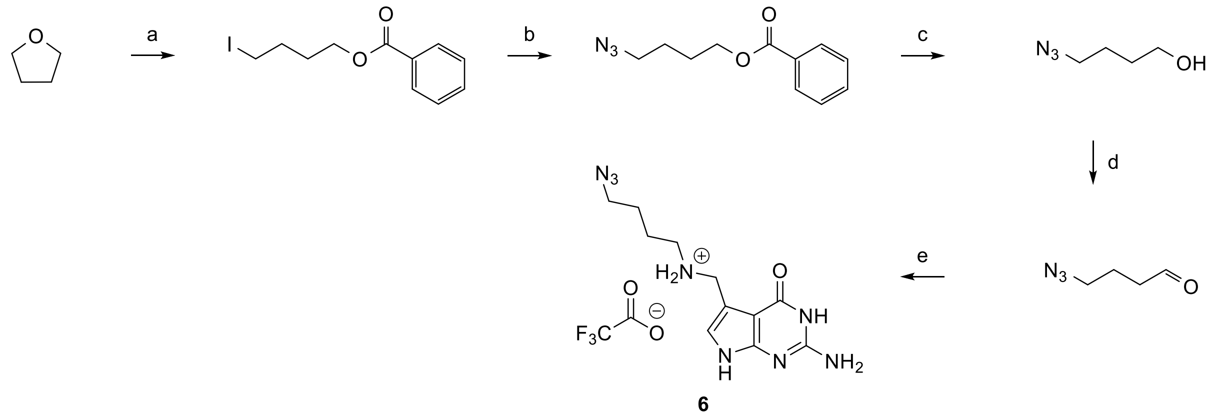

Scheme 5: Synthesis of compound **6**. a) NaI (1 eq), benzoyl chloride (1 eq), ACN, RT, overnight, 90%. b) NaN_3 (1.5 eq), DMF, 0°C – RT, overnight, quantitative. c) LiOH (1.2 eq), THF/H_2O/MeOH (10/4/1.1), RT, overnight, 95%. d) Dess-Martin periodinane (1.3 eq), CH_2Cl_2, RT, 1 h, 86%. e) $preQ_1$ (1.1 eq), $(CH_3)_4NHB(OAc)_3$ (3.1 eq), HOAc, DMF, RT, 5 h, 46%.

5.1. 4-Iodobutyl benzoate[1]

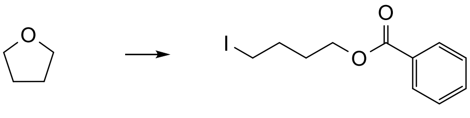

To a cooled solution of sodium iodide (18.4 g, 123 mmol) in tetrahydrofuran (10 ml, 123 mmol) and acetonitrile (5 ml), benzoyl chloride (14.8 ml, 123 mmol) was added in one portion. The reaction mixture was stirred in the dark overnight. After dilution of the reaction mixture with water and ether, the organic layers were separated and the aqueous layer was extracted three times with ether. The combined organic layers were washed with saturated $NaHSO_3$, Na_2CO_3 and dried over $MgSO_4$. The solvent was removed in vacuo to give the product as colorless oil.

Yield: 33.7 g (90%) colorless oil

^{1}H NMR (300 MHz, $CDCl_3$): δ 1.86-1.96 (m, 4H, 2x H_2-C); 3.22 (t, 2H, H_2-CO); 4.32 (t, 2H, H_2-CI); 7.42 (t, 2H, H-C(arom.)); 7.52 (m, 1H, H-C(arom.)); 8.02 (m, 2H, H-C(arom.)) ppm.

5.2. 4-Azidobutyl benzoate[1]

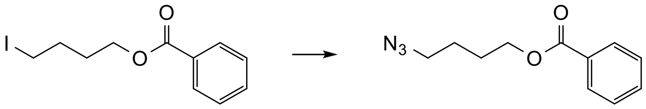

Sodium azide (5.89 g, 90.4 mmol) was added in portions to a solution of 4-iodobutyl benzoate (18.4 g, 60.2 mmol), in dimethylformamide (90 ml) in an ice bath in the dark. The reaction mixture was allowed to stir overnight and was subsequently diluted with ether and water. The organic layer was separated and the aqueous layer was further extracted with ether three times. The combined organic layers were concentrated in vacuo and the concentrate was diluted with hexane, washed with water and saturated NaCl, and dried over $MgSO_4$. The solvent was removed in vacuo to give the product as colorless oil.

Yield: 13.2 g (quantitative) colorless oil

^{1}H NMR (300 MHz, $CDCl_3$): δ 1.68-1.79 (m, 4H, 2x H_2-C); 3.27 (t, 2H, H_2-CN_3); 4.28 (t, 2H, H_2-CO); 7.37 (m, 2H, H-C(arom.)); 7.47 (m, 1H, H-C(arom.)); 8.00 (m, 2H, H-C(arom.)) ppm.

5.3. 4-Azidobutanol[1]

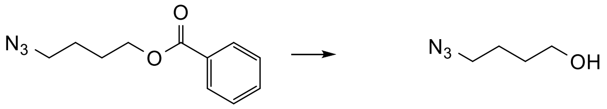

Lithium hydroxide (3.10 g, 74 mmol) was added to a solution of 4-azidobutyl benzoate (13.5 g, 61.6 mmol) in tetrahydrofuran (40 ml), water (16 ml) and methanol (4.4 ml). The reaction mixture was stirred overnight and subsequently diluted with water and ether. The layers were separated and the aqueous layer was further extracted with ether three times. The combined organic layers were washed with saturated NaCl and dried over $MgSO_4$. The solvent was removed in vacuo to give the product as yellow oil.

Yield: 6.73 g (95%) yellow oil

^{1}H NMR (300 MHz, $CDCl_3$): δ 1.72-1.56 (m, 4H, 2x H_2-C); 1.88 (s, 1H, OH); 3.31 (t, 2H, H_2-CN_3); 3.65 (t, 2H, H_2-COH) ppm.

5.4. 4-Azidobutanal

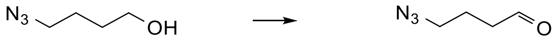

4-Azidobutanol (830 mg, 7.20 mmol) was dissolved in dry CH_2Cl_2 (70 ml) and Dess-Martin periodinane (3.97 g, 9.36 mmol) was added. The white suspension was stirred at room temperature under argon atmosphere. After 1 hour the mixture was diluted with dichloromethane, washed with saturated $Na_2S_2O_3$, $NaHCO_3$ and NaCl, dried over Na_2SO_4 and concentrated in vacuo. The crude product was purified by column chromatography on silica gel (diethyl ether/hexane, 25:75).

Yield: 697 mg (86%)

TLC (diethyl ether/hexane, 25:75): R_f = 0.21

^{1}H NMR (300 MHz, $CDCl_3$): δ 1.93 (m, 2H, H_2-C(2)); 2.60 (m, 2H, H_2-C(3)); 3.38 (m, 2H, H_2-C(1)); 9.82 (s, 1H, H-CO) ppm.

5.5. 7-((4-Azidobutyl)aminomethyl)-7-deazaguanine trifluoroacetate 6

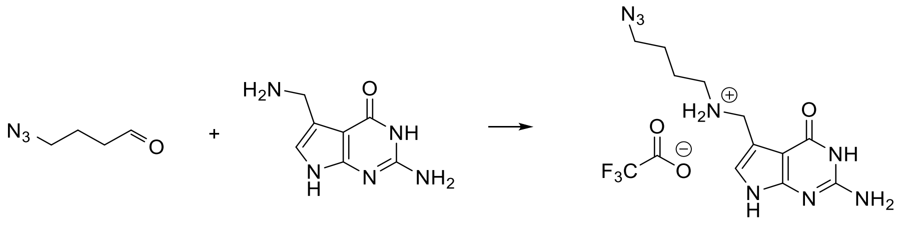

6

4-Azidobutanal (305 m, 2.70 mmol) was dissolved in dimethylformamide (30 ml) and $preQ_1$ trifluoroacetate (871 mg, 2.97 mmol) was added. After the addition of tetramethylammonium triacetoxyborohydride (2.21 g, 8.40 mmol) and acetic acid (154 µl) the solution was stirred for 5 hours at room temperature under argon atmosphere. The reaction mixture was concentrated in vacuo and the crude product was purified by reversed phase chromatography on an ÄKTA prime plus system (LiChroprep® RP-18 (40 – 63 µm), 1% trifluoroacetic acid/acetonitrile, 100:0 – 70:30, 360 ml, flow rate 3 ml/min).

Yield: 486 mg (46%) of **6** as beige solid

TLC (RP, acetonitrile/TFA(1%), 1:1): R_f = 0.66

ESI-MS (m/z): $[M+H]^+$ calculated: [277.31]; found: [277.07]

^{1}H NMR (300 MHz, d_6-DMSO): δ 1.60 (m, 4H, H_2-C(2',3')); 2.98 (m, 2H, H_2-C(4')); 3.37 (m, 2H, H_2-C(1')); 4.16 (m, 2H, H_2-C(7)); 6.34 (s, 2H, H_2-N(2)); 6.82 (s, 1H, H-C(8)); 8.94 (m, 2H, $^+H_2$-NC(7)); 10.9 (s, 1H, H-N(9)); 11.3 (s, 1H, H-N(1)) ppm.

6. Synthesis of compound 7

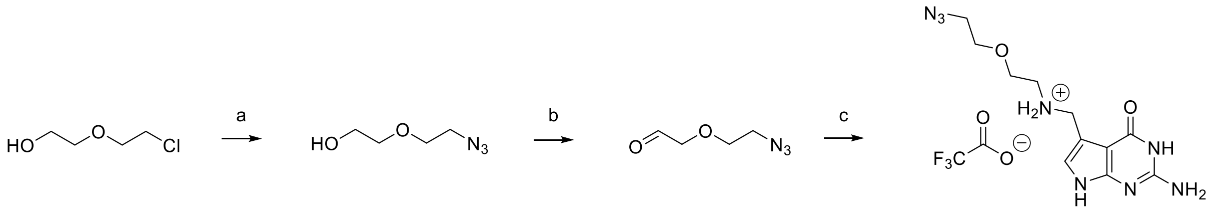

7

Scheme 6: Synthesis of compound **7**. a) NaN_3 (2 eq), H_2O, reflux, 21 h, 98%. b) Dess-Martin periodinane (1.3 eq), CH_2Cl_2, RT, 1 h, 88%. c) preQ$_1$ (1.1 eq), $(CH_3)_4NHB(OAc)_3$ (1.5 eq), HOAc, DMF, RT, 5 h, 37%.

6.1. 2-(2-Azidoethoxy)ethanol

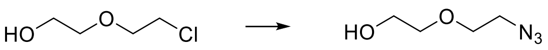

2-(2-Chloroethoxy)ethanol (8.47 ml, 80 mmol) was dissolved in ater (34 ml) and sodium azide (10.4 g, 160 mmol) was added. The reaction mixture was refluxed for 21 hours and then cooled to room temperature. The solution was extracted six times with 50 ml dichloromethane, dried over $MgSO_4$ and evaporated.

Yield: 10.3 g (98%) colourless oil

TLC (ethyl acetate/MeOH, 9:1): R_f = 0.60

^{1}H-NMR (300 MHz, $CDCl_3$): δ 2.37 (t, 1H, OH); 3.38 (t, 2H, H_2-C(2)); 3.58 (t, 2H, H_2-C(3)); 3.66 (t, 2H, H_2-C(4)); 3.69 - 3.75 (m, 2H, H_2-C(1)) ppm.

6.2. 2-(2-Azidoethoxy)acetaldehyde

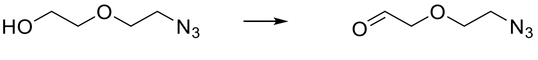

2-(2-Azidoethoxy)ethanol (337 mg, 2.57 mmol) was dissolved in dichloromethane (50 ml) and Dess-Martin periodinane (1.42 g, 3.34 mmol) was added portion wise. The solution was stirred at room temperature under argon for 1 hour, filtered through a celite pad and concentrated in vacuo. The crude product was purified by column chromatography on silica gel (diethyl ether/pentane, 1:2).

Yield: 293 mg (88%) colourless oil

TLC (diethyl ether /pentane, 1:2): R_f = 0.14

^{1}H-NMR (300 MHz, $CDCl_3$): δ 3.47 (t, 2H, H_2-C(3)); 3.74 (t, 2H, H_2-C(4)); 4.16 (s, 2H, H_2-C(2)); 9.73 (s, 1H, H-C(1)) ppm.

6.3. 7-((2-(2-Azidoethoxy)ethyl)aminomethyl)-7-deazaguanine trifluoroacetate 7

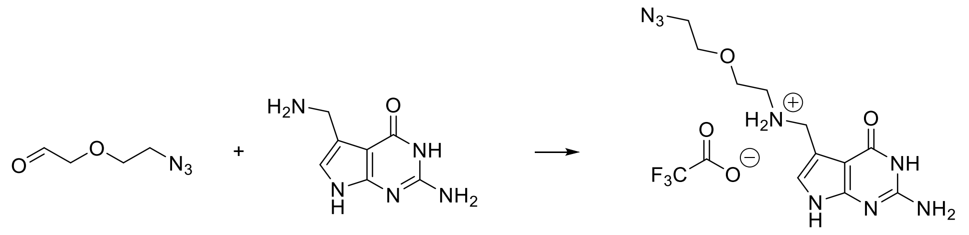

7

2-(2-Azidoetoxy)acetaldehyde (60 mg, 0.47 mmol) was dissolved in dimethylformamide (5 ml) and preQ_1 trifluoracetate (150 mg, 0.51 mmol) was added. Then tetramethylammonium triacetoxyborohydride (184 mg, 0.70 mmol) and acetic acid (30 µl) were added dropwise. The mixture was stirred at room temperature under argon for 5 hours, evaporated and dried under high vacuum. The crude product was purified by reversed phase chromatography on an ÄKTA prime plus system (LiChroprep® RP-18 (40 – 63 µm), 1% trifluoroacetic acid/acetonitrile, 100:0 – 70:30, 360 ml, flow rate 4 ml/min).

Yield: 69 mg (37%) of **7** as beige solid

ESI-MS (m/z): $[M+H]^+$ calculated: [293.15], found: [293.16]

^{1}H NMR (600 MHz, DMSO-d_6): δ 3.16 (t, 2H, H_2-C(1′)); 3.46 (t, 2H, H_2-C(4′)); 3.62 (t, 2H, H_2-C(3′)); 3.70 (t, 2H, H_2-C(2′)); 4.20 (t, 2H, H_2-C(7′)); 6.38 (s, 2H, H_2-N(2)); 6.82 (s, 1H, H-C(8)); 9.10 (s, 2H, H_2^+-N(7′)); 10.94 (s, 1H, H-N(1); 11.29 (s, 1H, H-N(9)) ppm.

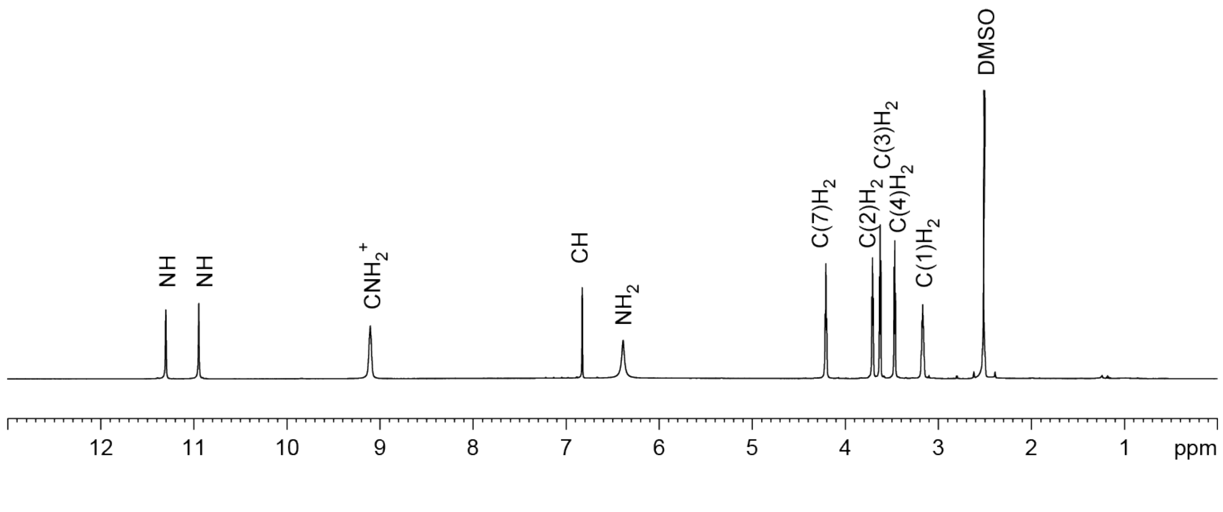

7. Synthesis of compound 8

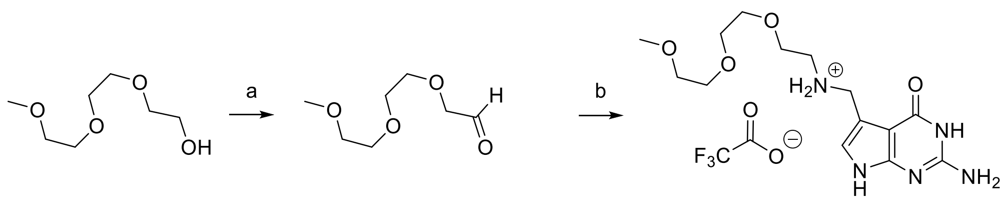

8

Scheme 7: Synthesis of compound **8**. a) Dess-Martin periodinane (1.15 eq), CH_2Cl_2, RT, 3 h, 73%. b) $preQ_1$ (1.1 eq), $(CH_3)_4NHB(OAc)_3$ (1.9 eq), HOAc, DMF, RT, 5 h, 21%.

7.1. 2-(2-(2-Methoxyethoxy)ethoxy)acetaldehyde

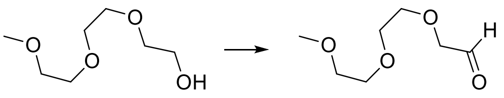

Dess-Martin periodinane (1000 mg, 2.35 mmol)was suspended in dry dichloromethane (20 ml) and triethylene glycol monomethyl ether (336 mg, 2.05 mmol) was added. The suspension was stirred at room temperature for 3 hours, filtered through a celite pad and evaporated.

Yield: 241 mg (73%)

^{1}H-NMR (300 MHz, d_6-DMSO): δ 3.38 (3H, H_3-C); 3.56 (t, 2H, H_2-C); 3.65 (t, 2H, H_2-C); 3.71 – 3.75 (m, 4H, H_2-C); 4.16 (2H, H_2-C(2)); 9.73 (1H, H-CO) ppm.

7.2. 7-(5,8,11-trioxa-2-azadodecyl)-7-deazaguanine trifluoroacetate 8

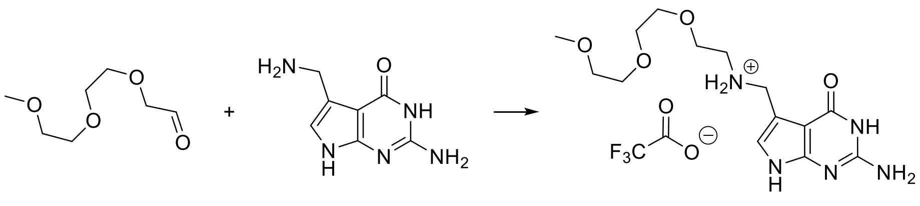

8

2-(2-(2-Methoxyethoxy)ethoxy)acetaldehyde (22 mg, 0.136 mmol) was dissolved in dry dimethylformamide (1 ml) and stirred at room temperature. Then, tetramethylammonium triacetoxyborohydride (66 mg, 0.253 mmol) and acetic acid (7 µl) were added successively. $PreQ_1$ trifluoroacetate (43 mg, 0.146 mmol) was dissolved in dry dimethylformamide (1 ml) and

was added dropwise to the reaction mixture. The solvents were evaporated after 5 hours and the crude product was purified by reversed phase column chromatography on an ÄKTA prime plus system (LiChroprep® RP-18 (40 – 63 µm), 1% trifluoroacetic acid/acetonitrile, 100:0 – 70:30, 360 ml, flow rate 4 ml/min).

Yield: 12.5 mg (21%) of **8** as yellow solid

HR-ESI-MS (m/z): $[M+H]^+$ calculated: [326.1823]; found: [326.1783]

^{1}H NMR (300 MHz, d_6-DMSO): δ 3.14 (2H, H_2-C); 3.23 (2H, H_2-C(7′)); 3.42 (2H, H_2-C); 3.52 (2H, H_2-C); 3.55 (5H, H_3-C, H_2-C); 3.66 (2H, H_2-C); 4.19 (2H, H_2-C); 6.31 (2H, H_2-N(2)); 6.82 (1H, H-C(8)); 9.01 (2H, H_2-N(7′)); 10.85 (1H, H-N(1)); 11.27 (1H, H-N(9)) ppm.

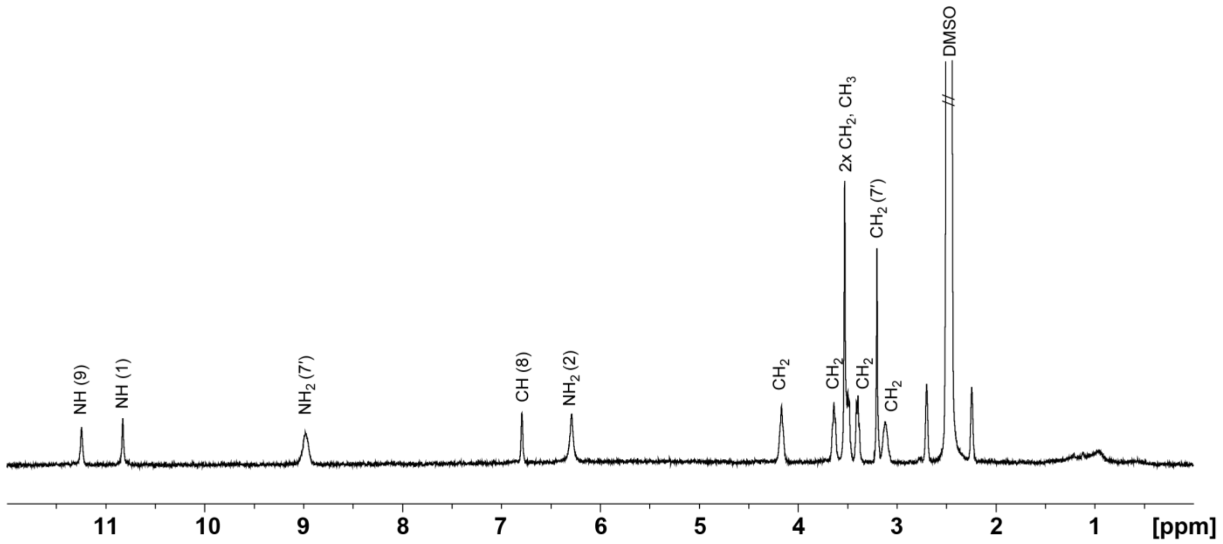

8. Synthesis of compound 9

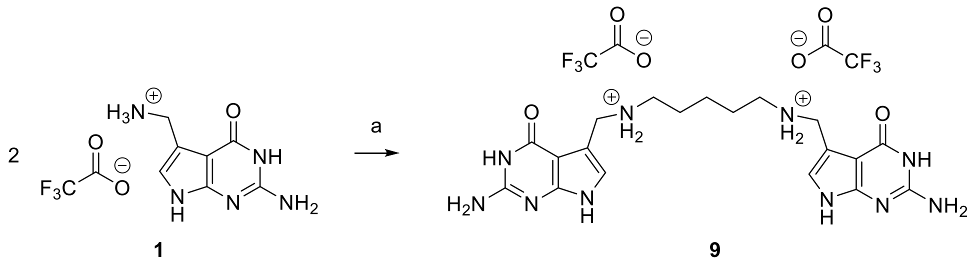

Scheme 8: Synthesis of compound **9**. a) glutaraldehyde (0.8 eq, 50wt%), $(CH_3)_4NHB(OAc)_3$ (3.2 eq), HOAc, DMF, RT, 4 h, 42%.

8.1. Pentyl-linked preQ$_1$ dimer 9

(2-(3-(2-amino-4-oxo-4,7-dihydro-3H-pyrrolo[2,3-d]pyrimidin-5-yl)propyl)isoindoline-1,3-dione) **9**

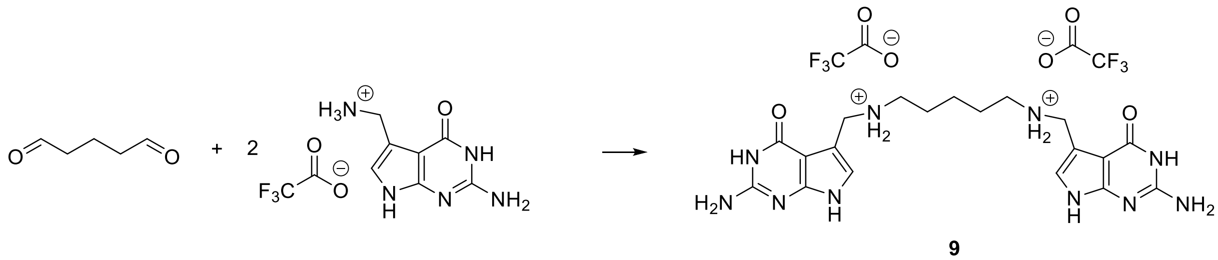

To a solution of preQ$_1$ trifluoroacetate (310 mg, 1.06 mmol) in dimethylformamide (5 ml), glutaraldehyde (84 mg, 50wt%, 0.423 mmol) was added. After the addition of tetramethylammonium triacetoxyborohydride (446 mg, 1.69 mmol) and acetic acid (114 µl) the orange solution was stirred for 4 hours at room temperature. The solvent was evaporated and the crude product was purified by reversed phase chromatography on an ÄKTA prime plus system (LiChroprep® RP-18 (40 – 63 µm), 1% trifluoroacetic acid/acetonitrile, 100:0 – 70:30, 480 ml, flow rate 4 ml/min).

Yield: 144 mg (42%) of **9** as white solid

TLC (RP, acetonitrile/water, 1:1): $R_f = 0.68$

ESI-MS (m/z): $[M+H]^+$ calculated: [427.48]; found: [427.30]

^{1}H NMR (300MHz, d$_6$-DMSO): δ 1.61 (m, 6H, H_2-C (2'-4'); 3.43 (m, 4H, H_2-C(1', 5')); 4.27 (s, 4H, H_2-C(7')); 6.34 (s, 4H, H_2-N(2)); 6.85 (s, 2H, H-C(8)); 9.68 (s, 4H, H_2^+-N(7')); 10.79 (s, 2H, H-N); 11.36 (s, 2H, H-N) ppm.

9. Solid-phase synthesis of oligonucleotides

Oligoribonucleotides were synthesized by solid-phase RNA synthesis. Standard phosphoramidite chemistry using 2'-*O*-TOM or 2'-O-*t*BDMS ribonucleoside phosphoramidite building blocks (ChemGenes, Sigma Aldrich) and control pore glass (CPG) supports (ChemGenes, 1000 Å, 36.8 µmol/g) was applied (2,3). 2-Aminopurine nucleoside phosphoramidite was purchased from ChemGenes. All oligonucleotides were synthesized on ABI 392 or 394 Nucleic Acid Synthesizers following standard methods: detritylation (80 s) with dichloroacetic acid/1,2-dichloroethane (4/96); coupling (2.0 min) with phosphoramidites/acetonitrile (0.1 M x 130 µl) and benzylthiotetrazole/ acetonitrile (0.3 M × 360 µl); capping (3 × 0.4 min, Cap A/Cap B = 1/1) with Cap A: THF/phenoxyacetic anhydride (95/5) and Cap B: 10% 1-Methylimidazole in THF/sym-collidine (8/1); oxidation (1.0 min) with I_2 (20 mM) in THF/pyridine/H_2O (35/10/5). The solutions of amidites, tetrazole, and acetonitrile were dried over activated molecular sieves (3 Å) overnight.

10. Deprotection of oligonucleotides

The solid support was treated each with ammonium hydroxide (28-30%, 0.5 ml) and methylamine in water (40%, 0.5 ml) for 5 h at room temperature. The supernatant was removed and the solid support was washed 3 × with ethanol/water (1/1, v/v). The supernatant and washes were combined and evaporated to dryness. To remove the 2'-silyl protecting groups the resulting residue was treated with tetrabutylammonium fluoride trihydrate (TBAF·3H_2O) in THF (1 M, 2 ml) at 37°C overnight. The reaction was quenched by the addition of triethylammonium acetate (TEAA) (1 M, pH 7.4, 2 ml). The volume of the solution was reduced and the solution was desalted with a size exclusion column (GE Healthcare, HiPrep 26/10 Desalting; 2.6 × 10 cm; Sephadex G25) eluting with H_2O, the collected fraction was evaporated to dryness and dissolved in 1 ml H_2O. Analysis of the crude RNA after deprotection was performed by anion-exchange chromatography on a Dionex DNAPac PA-100 column (4 mm × 250 mm) at 80°C. Flow rate: 1 ml/min, eluant A: 25 mM Tris·HCl (pH 8.0), 6 M urea; eluant B: 25 mM Tris·HCl (pH 8.0), 0.5 M $NaClO_4$, 6 M urea; gradient: 0-60% B in A within 60 min, UV detection at 260 nm.

11. Purification of RNA

Crude RNA products were purified on a semipreparative Dionex DNAPac PA-100 column (9 mm × 250 mm) at 80°C with flow rate 2 ml/min. Fractions containing RNA were loaded on a C18 SepPak Plus cartridge (Waters/Millipore), washed with 0.1 M $(Et_3NH)^+HCO_3^-$, H_2O and

eluted with H_2O/CH_3CN (1/1). RNA containing fractions were lyophilized. Analysis of the quality of purified RNA was performed by anion-exchange chromatography with same conditions as for crude RNA. The molecular weight was confirmed by LC-ESI mass spectrometry. Yield determination was performed by UV photometrical analysis of oligonucleotide solutions.

12. Mass spectroscopy of RNA

All experiments were performed on a Finnigan LCQ Advantage MAX ion trap instrument connected to an Amersham Ettan micro LC system. RNA sequences were analyzed in the negative-ion mode with a potential of -4 kV applied to the spray needle. LC: Sample (200 pmol RNA dissolved in 30 µl of 20 mM EDTA solution; average injection volume: 30 µl); column (Waters XTerraMS, C18 2.5 µm; 1.0 × 50 mm) at 21°C; flow rate: 30 µl/min; eluant A: 8.6 mM TEA, 100 mM 1,1,1,3,3,3-hexafluoroisopropanol in H_2O (pH 8.0); eluant B: methanol; gradient: 0–100% B in A within 30 min; UV detection at 254 nm.

13. Determination of ligand binding affinities (K_D)

All fluorescence experiments were carried out on a Cary Eclipse spectrometer (Varian, Palo Alto, USA) equipped with a peltier block, a magnetic stirring device, and a RX2000 stopped-flow apparatus (Applied Photophysics Ltd., Leatherhead, UK). 2-Aminopurine labeled RNA samples (0.5 µM) were prepared in a total volume of 1000 µl of buffer (50 mM MOPS, 100 mM KCl, 2 mM $MgCl_2$, pH 7.5) (4). The samples were heated to 90°C for 2 minutes, allowed to cool to room temperature and held at 20°C in the peltier controlled sample holder in a quartz cuvette equipped with a small stir bar. Then, ligand was manually pipetted in 1µL aliquots in a way not to exceed a total volume increase of 2%. The solution was stirred during each titration step and allowed to equilibrate for at least 10 minutes before data collection. Spectra were recorded from 320 to 500 nm using the following instrumental parameters: excitation wavelength, 308 nm; increments, 1 nm; scan rate, 120 nm/min; slit widths, 10 nm. The apparent binding constants K_D were determined by following the increase in fluorescence after each titration step via integration of the area between 330 and 450 nm. Changes in fluorescence ($F - F_0$) were normalized to the maximum fluorescence measured in the absence of ligand. The measurement for each titration step was repeated at least two times and the mean of the normalized fluorescence intensity was plotted against the ligand concentration. Data were fit using a K_D quadratic equation solution for 1:1 stoichiometry.

$$\frac{(F-F_0)}{(F_f-F_0)}=\frac{K_D+[RNA]_{total}+[ligand]_{total}\pm\sqrt{(K_D+[RNA]_{total}+[ligand]_{total})^2-4[RNA]_{total}[ligand]_{total}}}{2[RNA]_{total}}$$

F (fluorescence), F_0 (initial fluorescence), F_f (final fluorescence); $[RNA]_{total}$ (total concentration of RNA), $[ligand]_{total}$ (total concentration of added ligand for each titration step).

14. Determination of ligand binding kinetics (k_{obs} and k_{on})

Rate constants *k* for individual riboswitch variants were measured under pseudo-first-order conditions with ligand in excess over RNA (5). Stock solutions were prepared for each 2-aminopurine labeled RNA variant (concentration c_{RNA} = 0.6 μM in 50 mM MOPS pH 7.5, 100 mM KCl, 2 mM $MgCl_2$) and for each ligand (concentration c_{ligand} = 1.2 to 13.2 μM in 50 mM MOPS pH 7.5, 100 mM KCl, 2 mM $MgCl_2$). Mixing equal volumes of these stock solutions via the stopped-flow apparatus resulted in a final concentration of 0.3 μM RNA and of 0.6 to 6.6 μM ligand. Spectra were recorded at 20 °C using the following instrumental parameters: excitation wavelength, 308 nm; emission wavelength, 372 nm; increment of data point collection, 0.05 s; slit widths, 10 nm.

The stopped-flow fluorescence data were fit to a single-exponential equation:

$$F = A_1+A_2e^{-k't}$$

A_1 corresponds to final fluorescence; $A_2e^{-k't}$ corresponds to the change in fluorescence over time (t) at the observed rate k'. The measurement for each concentration was repeated at least two times and the mean of the observed rates k' was plotted against ligand concentration to obtain the rate constant k from the slope of the plot. The final rate constant *k* value is an arithmetic mean, determined from two independent stopped-flow measurements. All data processing was performed using *Kaleidagraph* software (Synergy Software, Reading, UK).

15. Cellular assays (IC_{50})

Synthetic class I and class II $preQ_1$ riboswitch encoding DNAs with *EcoR*I and *Sph*I overhangs were purchased from IDT (Integrated DNA Technologies) and cloned into *EcoR*I/*Sph*I sites of a pQE-70 bacterial expression vector (Qiagen) upstream to an eGFP reporter gene. Thus, the introduced riboswitch sequences replace the vector-encoded ribosomal binding site and provide the Shine-Dalgarno sequence required for translation. The plasmids were transformed

into $preQ_1$-deficient *E.coli* B105 cells (6). Bacterial cultures were grown to an OD_{600} of ~0.5 in LB medium (1% tryptone, 0.5% yeast extract, 0.5% NaCl, 10 mM $MgSO_4$, pH 7) before induction of reporter transcription by addition of 0.8 mM isopropyl-β-D-thiogalactopyranoside (IPTG). At the same time, ligands were added at different concentrations (0.1 µM – 7.5 mM). eGFP production was monitored by fluorescence measurement of 150 µl expression culture at 0 and 6 hours incubation using a Mithras LB 940 microplate reader (Berthold Technologies) and 96-well black clear bottom plates (Costar) with an excitation wavelength of 485 nm and an emission wavelength of 535 nm. The fluorescence intensities were corrected for background fluorescence of LB medium and normalized to the cell density determined at 600 nm. All data was log transformed and the IC_{50} value was determined with a fit to a four-parameter logistic equation.

$$\mathrm{Y} = \mathrm{B}\,\frac{\mathrm{C}-\mathrm{B}}{1+10^{((\log(\mathrm{IC50}))*\mathrm{HillSlope})}}$$

Y corresponds to % inhibition; B corresponds to the bottom plateau of % inhibition; C corresponds to the top plateau of % inhibition; X corresponds to the log of ligand concentration; HillSlope corresponds to the steepness of the curve; log(IC50) corresponds to the log of the half maximal inhibitory concentration. The final IC_{50} value was derived from at least two independent measurements. All data processing was performed using *GraphPad Prism 5* software (GraphPad Software, La Jolla, USA).

16. References

1. Bates,R.W.; Dewey,M.R., (2009) A formal synthesis of swainsonine by gold-catalyzed allene cyclization. *Org. Lett.*, **11**, 3706–3708.

2. Micura,R. (2002) Small interfering RNAs and their chemical synthesis. *Angew. Chem. Int. Ed.*, **41**, 2265–2269.

3. Pitsch,S., Weiss,P.A., Jenny,L., Stutz,A. and Wu,X. (2001) Reliable chemical synthesis of oligoribonucleotides (RNA) with 2′-O-[(triisopropylsilyl)oxy]methyl (2′-O-tom)-protected phosphoramidites. *Helv. Chim. Acta*, **84**, 3773–3795.

4. Rieder,R., Lang,K., Graber,D. and Micura,R. (2007) Ligand-induced folding of the adenosine deaminase A-riboswitch and implications on riboswitch translational control. *ChemBioChem*, **8**, 896–902.

5. Rieder,U., Kreutz,C. and Micura,R. (2010) Folding of a transcriptionally acting $preQ_1$ riboswitch. *Proc. Natl. Acad. Sci. U.S.A.*, **107**, 10804–10809.

6. Gaur,R. and Varshney,U. (2005) Genetic analysis identifies a function for the queC (ybaX) gene product at an initial step in the queuosine biosynthetic pathway in Escherichia coli. *J. Bacteriol.*, **187**, 6893–6901.

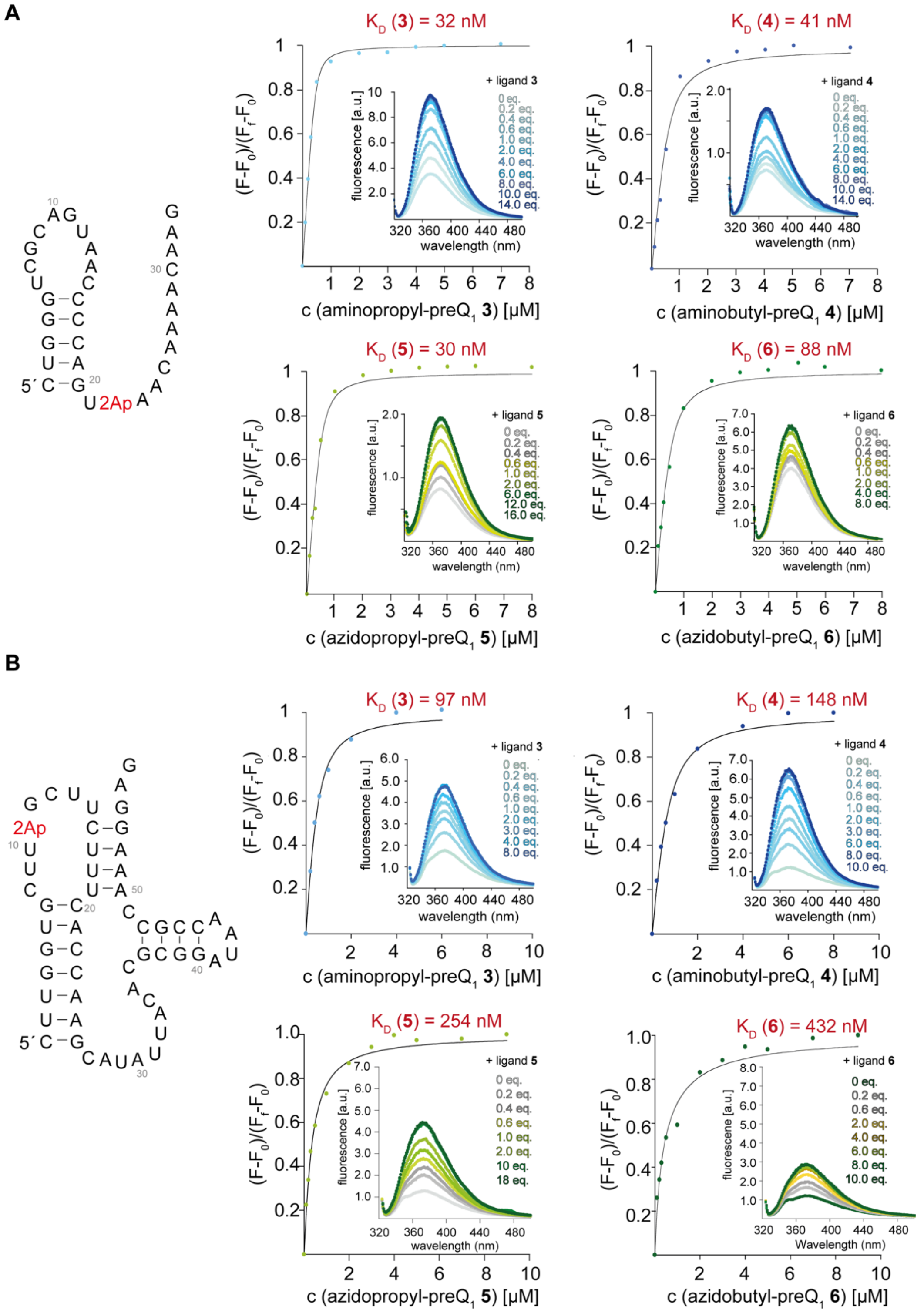

Supporting Figure S1. Fluorescence changes upon titration of preQ1-I (U22Ap) (**A**) and preQ1-II (A11Ap) (**B**) riboswitches with different concentrations of preQ1 analogs (**3**, **4**, **5**, and **6**) for the determination of K_D-values. Normalized fluorescence intensities of the U22Ap variant were plotted as a function of preQ1 concentrations. Changes in fluorescence (F-F0) were normalized to the maximum fluorescence measured in saturating concentrations of preQ1. The graph shows the best fit to a single-site binding model (see Supplementary Methods). The insets show fluorescence emission spectra (λ_{ex} = 308 nm) from 320 to 500 nm for each ligand concentration. For conditions see Supplementary Methods. For an overview of K_D-values of all ligand anaolgs investigated in this study see Tables 1 and 2.

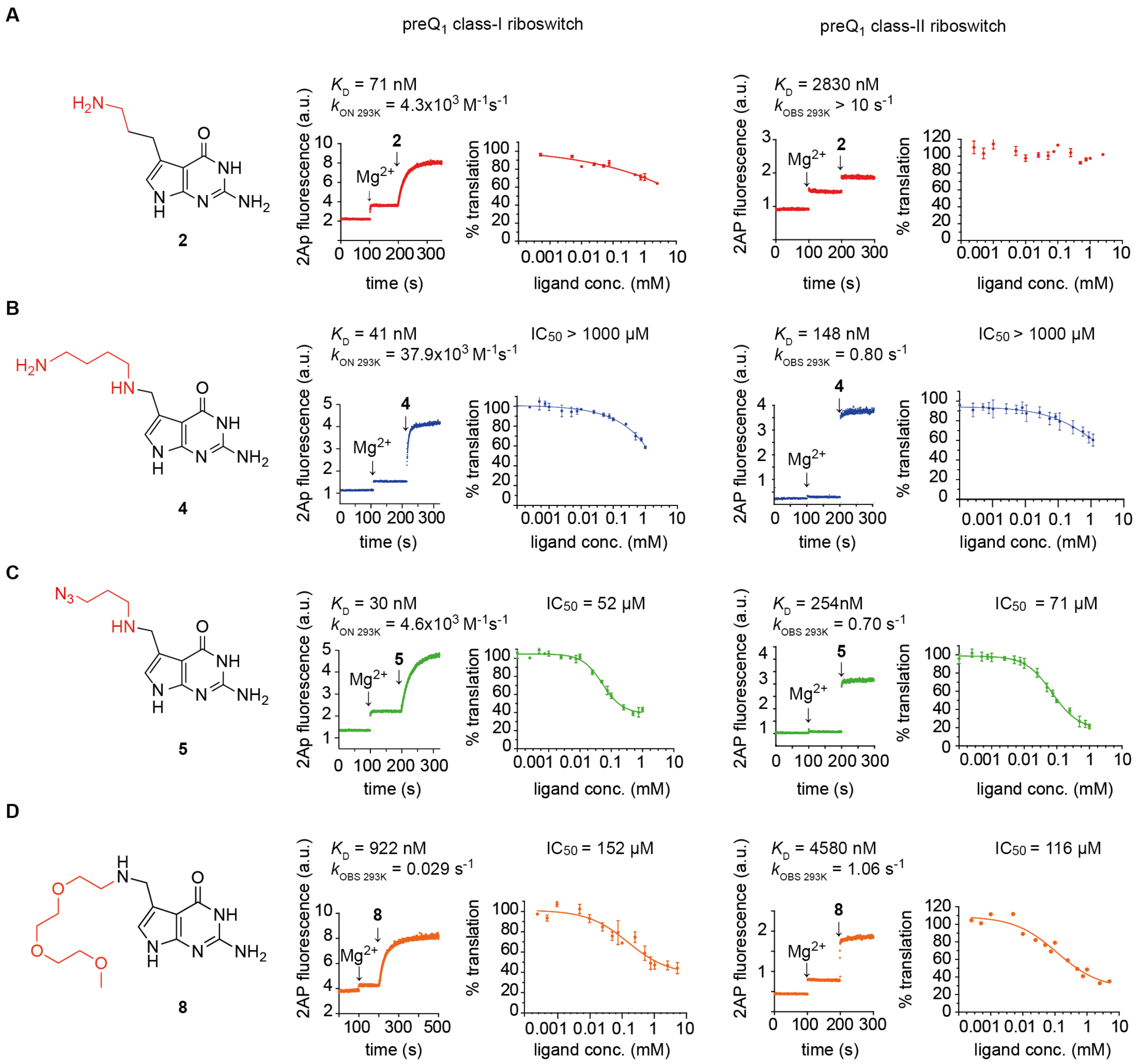

Supporting Figure S2. Comparison of *in vitro* and *in vivo* performance of $preQ_1$ class-I and -II riboswitches with modified ligand derivatives. **A**) Exemplary fluorescence time traces of Ap-labeled $preQ_1$ RNAs in response to Mg^{2+} and ligand analog **2** (conditions: 0.5 μM RNA, 100 mM KCl, 50 mM MOPS, pH 7.5, 293 K. Ligands: 2 mM $MgCl_2$, 5 μM compound **2**); affinities K_D were obtained from plots of normalized AP fluorescence intensities plotted as a function of ligand concentrations (for details see Supporting Figure S2); rate constants $k_{on(293)}$ of the *Tte* $preQ_1$ class-I riboswitch were obtained from plots of observed rates k_{obs} *vs* ligand concentrations (for details see Supporting Figure S3); binding to the *Spn* $preQ_1$-II riboswitch was independent of ligand concentration; for details of k_{obs} determination see Supporting Figure S4. Dose-dependent repression of gene expression: B105 *E. coli* cells transformed with constructs expressing eGFP under translational control of $preQ_1$ class-I and -II riboswitches were assayed for eGFP fluorescence in the presence of different $preQ_1$ **2** concentrations (0.10 μM to 5 mM). Data represent the mean of three biological replicates, with error bars indicating standard deviation. The data were fit with a four-parameter logistic function to derive the IC_{50} values as indicated. **B**) Same as (**A**), but for ligand derivative **4**. **C**) Same as (**A**), but for ligand derivative **5**. **D**) Same as (**A**), but for ligand derivative **8**.

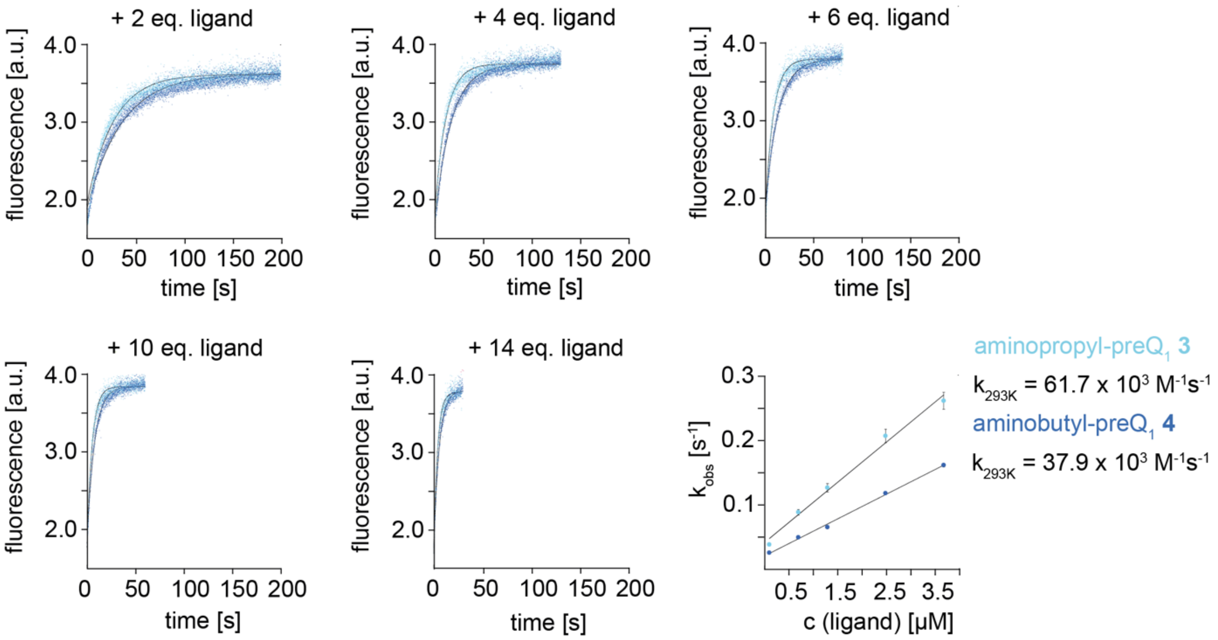

Supporting Figure S3. Stopped-flow fluorescence spectroscopy was used to monitor the kinetics of ligand preQ$_1$ binding to the *Tte* preQ$_1$ class-I riboswitch. Real time Ap fluorescence time traces of the *Tte* U22Ap variant at different concentrations of 3-aminopropyl- and 4-aminobutyl-modified preQ$_1$ **3** and **4** (solid line represents single-exponential curve fits); rate constants k_{293} from plots of observed rate k_{obs} versus ligand concentration (bottom right). c(RNA) = 0.3 µM, c($MgCl_2$) = 2 mM, 100 mM KCl, 50 mM MOPS, pH 7.5, 293 K. Ligand concentration c(ligand) as indicated.

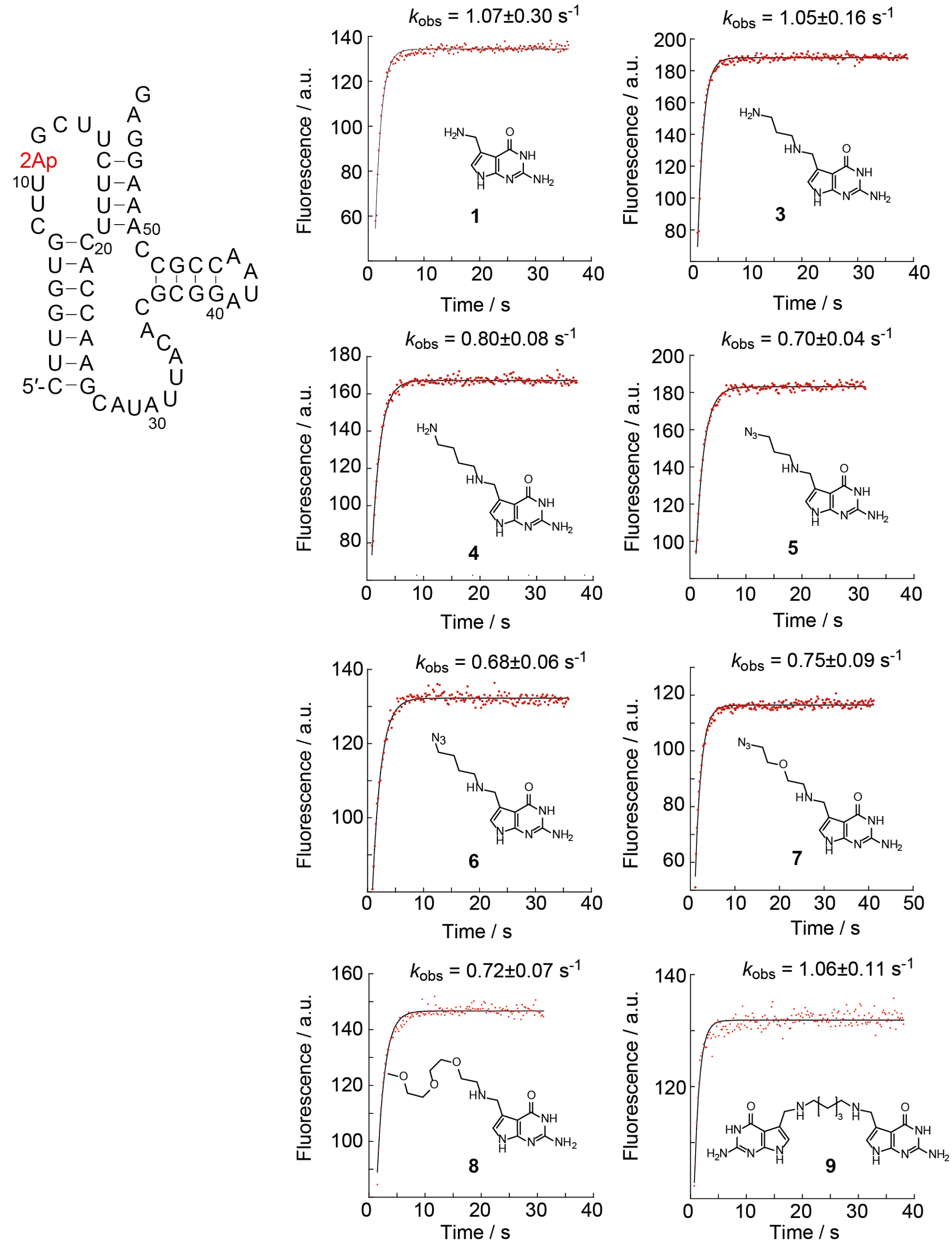

Supporting Figure S4. Stopped-flow fluorescence spectroscopy was used to monitor the kinetics of ligand preQ$_1$ binding to the *Spn* preQ$_1$ class-II riboswitch. Exemplary real time Ap fluorescence time traces of the *Spn* A11Ap variant in response to preQ$_1$ **1** and preQ$_1$ analogs **3** to **9** (solid line represents single-exponential curve fits); the observed rates k_{obs} were independent of ligand concentrations in the range tested (2 to 14-fold excess of ligand). c(RNA) = 0.3 µM, c($MgCl_2$) = 2 mM, 100 mM KCl, 50 mM MOPS, pH 7.5, 293 K. Ligand concentration c(ligand) = 1.8 µM.
